# SaDA: From Sampling to Data Analysis—An Extensible Open Source Infrastructure for Rapid, Robust and Automated Management and Analysis of Modern Ecological High-Throughput Microarray Data

**DOI:** 10.3390/ijerph120606352

**Published:** 2015-06-03

**Authors:** Kumar Saurabh Singh, Dominique Thual, Roberto Spurio, Nicola Cannata

**Affiliations:** 1 School of Biosciences and Veterinary Medicine, University of Camerino, Via Gentile III da Varano Camerino 62032, Italy; E-Mail: roberto.spurio@unicam.it; 2 Next Generation Bioinformatics s.r.l Camerino 62032, Italy; E-Mail: dom_thual@yahoo.fr; 3 School of Science and Technology, University of Camerino, Via Madonna delle Carceri Camerino 62032, Italy; E-Mail: nicola.cannata@gmail.com

**Keywords:** software, data management, microarrays, ecological assessment, environmental studies, LIMS, open source system

## Abstract

One of the most crucial characteristics of day-to-day laboratory information management is the collection, storage and retrieval of information about research subjects and environmental or biomedical samples. An efficient link between sample data and experimental results is absolutely important for the successful outcome of a collaborative project. Currently available software solutions are largely limited to large scale, expensive commercial Laboratory Information Management Systems (LIMS). Acquiring such LIMS indeed can bring laboratory information management to a higher level, but most of the times this requires a sufficient investment of money, time and technical efforts. There is a clear need for a light weighted open source system which can easily be managed on local servers and handled by individual researchers. Here we present a software named SaDA for storing, retrieving and analyzing data originated from microorganism monitoring experiments. SaDA is fully integrated in the management of environmental samples, oligonucleotide sequences, microarray data and the subsequent downstream analysis procedures. It is simple and generic software, and can be extended and customized for various environmental and biomedical studies.

## 1. Introduction

To gain an understanding of complex microbial environments, researchers have to assemble different type of information originating from heterogeneous sources. Every bit of information is valuable and should be made available whenever it is required. An exhaustive and clear protocol for data collection, processing and analysis is critical to achieve the projects end goals. The rapid increase in the discovery and application of molecular techniques constitutes important steps towards the detection and identification of microorganisms present in complex environmental samples. Identification and characterization of microorganisms is a key part of the management of environment and quality of life, tracing contaminants and troubleshooting problems such as pathogenesis or pollution indication [1]. Identification of an unknown species can help to assess whether it poses a safety concern or not. Generally, the process of bio-monitoring plays a crucial role in the execution of large scale projects which focus either on the protection of people’s health, safety of materials or dealing with environment quality. Big ecology related projects address very complicated issues [2]. They require generation and collection of data from a wide spectrum of sources, collaboration of people from different disciplines and the application of highly complex analytical approaches [3]. Moreover, information management is important especially when a large volume of complex data is collected. It becomes even more important when multiple organizations, at different geographical locations, are involved in the data collection procedures. It is absolutely essential that data are collected and managed using the methods recognized by scientific community. Existing software systems do not typically overlap the full pipeline as far as ecological collaborative projects are concerned. They are limited by data types, provide limited extensibility or require additional skills to handle data which are beyond the scope of experimental researchers. We developed Sampling to Data Analysis (SaDA) keeping in mind the requirements of individual laboratory involved in big collaborative projects. The system is a complete portal for ecology related high-throughput data management and analyses. Extra care was taken in designing database schema, information retrieval and in data presentation. SaDA is customizable and extensible to meet the needs of diverse research activities as the source code is freely available. The core of SaDA is based on model dependent development where abstract models of software systems are created and systematically transformed to its implementation [4]. The current version of SaDA supports data management and analysis protocols involved in fresh water monitoring systems utilizing microarray technology as a state-of-the-art technology. 

### State-of-Art Technology

To the best of our knowledge no freely available software is focused on microarray data management and analysis in terms of its ecological application, as in environmental monitoring. The aim of SaDA is to provide a streamlined work-flow from environmental sample collection and its downstream analysis. Microarray technology, however, is used here as a state-of-the-art technology to detect and quantify pathogens and other microorganisms in environmental freshwater samples. SaDA currently supports Agilent’s one channel oligonucleotide hybridization microarray technology. In addition, the downstream data analysis detects the presence or absence of signals, normalization and estimating cell counts from the micro-array signal intensities. SaDA is not a tool for gene expression analysis. Although existing software tools could meet some of the requirements of SaDA, none meet all of them in the form of a comprehensive, end-to-end platform available as open source and extensible system. Some tools have experienced only limited use. The state of art in Laboratory Information Management System (LIMS) provides all aspect of microarray data management and analysis, using advanced automation and precise robotic control. This type of integrated solutions ensures high quality and reliable data output and enables multiple projects to be managed in parallel. LIMS are mainly commercial and expensive software products. Few commercial systems lack clarity of open source systems, come at considerable costs, and requires high level of technical expertise to install and configure hardware, software and database and is not discussed in the present work. 

MARS [5] provides a comprehensive MIAME supportive suite for storing, retrieving, and analyzing multi-color microarray data. iLAP [6], based on a workflow driven modular architecture, was developed specifically to create and manage experimental protocols and to analyze and share laboratory data. Base [7] has been proved to be a comprehensive Microarray data repository, providing researchers an efficient data management and analysis tool. Labkey server [8] is a complete system with easy to use interfaces for specimen request submission across collaborators, allowing users to graphically define new data-types to fit in diverse datasets. It interacts dynamically with external data sources and supports developing custom interfaces using client libraries. GnomEx [9] provides comprehensive automated protocols for sample submission, sample tracking, billing, data management and analysis workflows for both Next Generation Sequencing (NGS) and Microarrays. LIMS Light System [10] has been developed to meet the demands of high-throughput primary data storage and data upload/retrieval. This system provides an efficient storage system with a Hierarchical Storage Management System and an Oracle relational database. OpenBis [11] is a flexible framework that has been adapted to be used for proteomics, high content screening and NGS projects. The SMITH [12] and WASP [13] system has been developed to meet the demands of NGS experiments and clinical tests. They provide embedded pipelines for the analysis of ChIP-Seq, RNA-Seq, miRNA-Seq and Exome-Seq experiments.There is also an extension with LIMS functionality to the popular Galaxy [14] workflow engine called Galaxy LIMS [15]. The system supports request submission, offers assistance during flow cell layout, and automatically launches Illumina’s CASAVA software [16] to perform de-multiplexing and delivery of the user-specific NGS raw files. Being integrated with Galaxy, the data are automatically available to be processed by analysis pipelines stored in Galaxy. SLIMS [17] is a sample management tool for genotyping laboratories. 

An open-source LIMS must be utilized to the specific infrastructure and the project needs of a research institution, which are in constant evolution. Therefore, the LIMS system must be modified accordingly. The effort required to tweak an existing LIMS and gain significant insight into its code base in order to be able to adjust it in a productive way must be compared with the effort of developing a new system. Large number of available LIMS software and tools substantiate the notion of creating a new system against tweaking an existing one. From this perspective, it seems that a simple LIMS has more chances of being shared than a more complex one. In any case, there is no LIMS that serves all needs in a simple turn-key solution. SaDA is not properly a LIMS based software solution but it provides a platform for model driven data management and analysis, and is flexible enough to include new features quickly and efficiently.

## 2. Material and Methods 

SaDA is a web application implemented in Ruby on Rails that runs on Phusion passenger application server and stores its data in a directory structure with in SaDA for quick file manipulations and also in a relational database engine for efficient and quick data querying and retrieval. The current version of SaDA is supported on Linux, UNIX, Mac OS and Microsoft Windows.

### 2.1. Implementation

**Figure 1:**
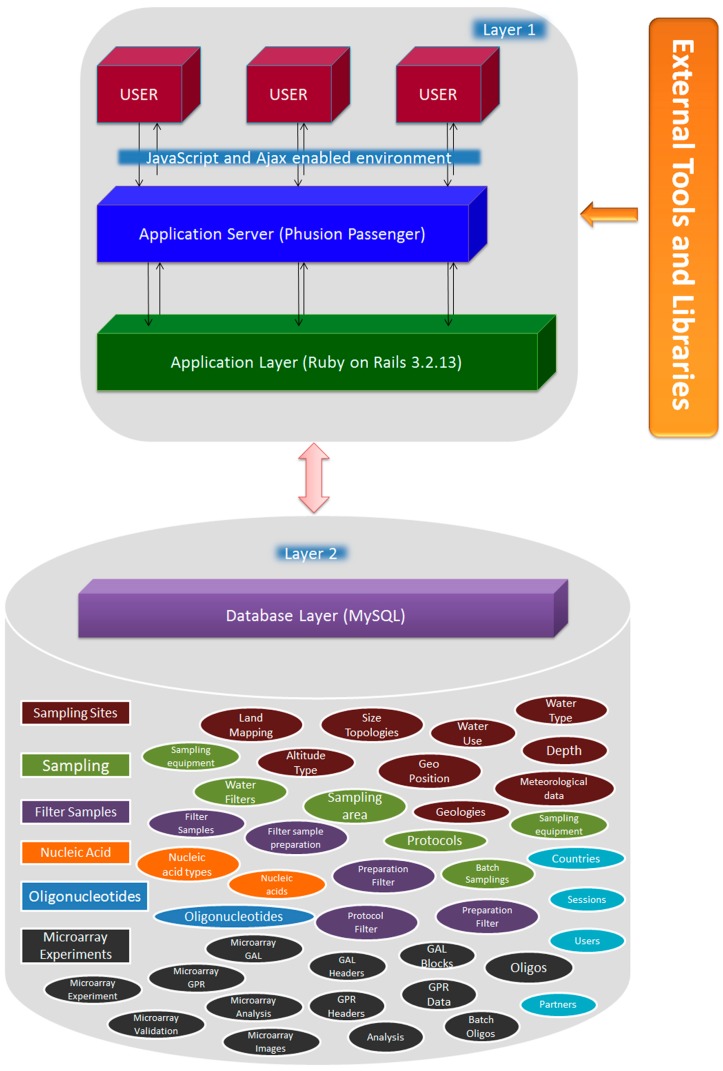
SaDA architecture: A multilayer architecture, comprising of layer 1 for data upload, processing and retrieval and a RDBMS storage layer with data access control, metadata storage for fast data retrieval. SaDA, in addition, has an external component for third party tools and libraries.

SaDA has been developed using the Ruby on Rails web application development framework, which is based on the Model-View-Controller (MVC) architectural pattern [18] of software development. MVC partition the application into multiple components. As described schematically in Figure 1, application input, output and processing (application server and application layer) are compartmentalized in two layers. Application’s core functionalities and database are encapsulated in layer 2, also described as “database layer” in Figure 1. The modular architecture of SaDA supports different features to be encapsulated as individual models, shown as ellipses Figure 1. Sharp rectangles are individual sections encompassing models with associations and relationships. For example, Sampling sites can have different features like land mapping, geographical positions, altitude type, water type & kind of water in use (for fresh water environments). These features are individual relations/tables associated with each other and also with features of different sections in the SaDA database. Database layer of SaDA is independent of input behavior and output data representation and forms the Model of MVC pattern. User interfaces and application server are components of layer 1. This part handles most of the input, output and query processing operations. It sends query to database and receives the data which in turn is displayed on SaDA user interfaces. The user interacts with the system solely through layer 1. The separation of the software layers allows freedom from the one-table-one-view paradigm. Therefore SaDA can display multiple interfaces of the same model allowing display of same data in multiple ways. Layer 1, in Figure 1, also includes an application server layer. The main job of this application layer is to support the construction of dynamic pages by handling the application’s program logic. Additionally, it also handles database connection and data transfers between client and application in a smooth and efficient way. External tools and libraries are used in SaDA to enhance some of the SaDA’s core functionalities described in later sections of the paper.

### 2.2. Experimental Design and Data Acquisition

Archiving detailed information on collection and analysis of samples is imperative for both efficient reporting of any study and for successive data analysis [19]. Collecting and storing these information is of utmost importance in terms of its application in high-throughput technologies. SaDA currently supports MySQL as a back end relational database management system (RDBMS). It can also support any other available RDBMS like SQLite and PostgreSQL. This kind of architecture permits multilevel security, as the application can be made secured at both application level and database level. SaDA provides a secure solution by implementing proper authentication control to each research participant. The database level security can be achieved by providing user level privileges. The system is web-based, which means that non-identifiable information is kept on a server and can be securely accessed on-line for queries or new submissions by authorized users via a web-browser. SaDA is simple and generic, and thus can be customized for various types of ecological as well as biological studies.

**Figure 2:**
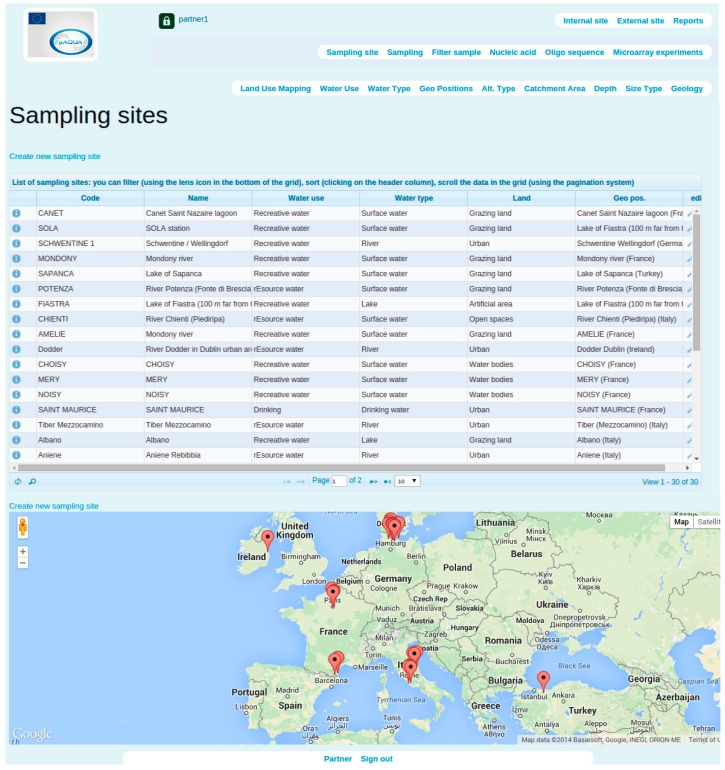
Display of *Sampling Sites* related data within SaDA.

### 2.3. The Features of SaDA

SaDA was originally developed on the guidelines provided by the MicroAQUA consortium [20]. The source code is now available to the public. Due to its modular framework (Figure 1), SaDA can be easily modified and applied to any environmental related projects. The current implementation of SaDA includes different modules storing data from sample collection, DNA/RNA extraction, oligonucleotides annotation, and microarrays raw data storage and validation results.

#### 2.3.1. Samples and Filter Tracking 

Users can track sample related information like quantity of samples and instrument utilized in sampling. From this point, the application assigns a unique identifier to the sample and provides the ability to be cross referenced later in other modules. Sampling sites, in addition, acts as one of the attributes of sampling procedure. Therefore SaDA integrates a Google Maps plug-in [21] for displaying sample collection sites (Figure 2) dynamically. Filter sample module saves information about the filter used to concentrate the collected water sample. A unique identifier specifies the filter associated with sampling and partner code. It carries information like pore size of the filter, number of filter tubes utilized and volume of water sample filtered.

#### 2.3.2. Management of project specific experimental data

SaDA supports oligonucleotide microarrays as a fresh water environment monitoring technology. SaDA includes data containers specific to the microarray experimental procedure and data analysis pipeline. Users can store information related to the procedures used to extract and quantify total RNA or DNA. Management of designed oligonucleotides is also possible in SaDA. The oligonucleotides module has an inbuilt taxonomy browser to assign taxonomy classification to the oligonucleotides. This particular feature helps in maintaining the classification of oligonucleotides which can be further categorized into hierarchical oligonucleotides.

#### 2.3.3. Microarray data management and analysis

A collection of microarray data can be viewed abstractly as a table with rows representing genes and columns representing various samples. Each position in the table describes the detection of a particular gene in a particular sample. We call this table a hybridization matrix. In SaDA, the information required to describe a microarray experiment can be divided conceptually into five logical parts which corresponds to raw and analyzed files. Raw data are images derived from scans of microarray following hybridization experiments. These images are analyzed to identify and quantify each spot of the array. There are at least three levels of data which are relevant to a microarray experiment:

- The scanned images (raw data) available in images sub-module.
- The quantitative outputs from the image analysis procedure (microarray quantitation matrices), available as GPR files that can be uploaded via Microarray .GPR section.
- The derived measurements (data matrices) that could be viewed from Microarray Analysis section.

**Figure 3:**
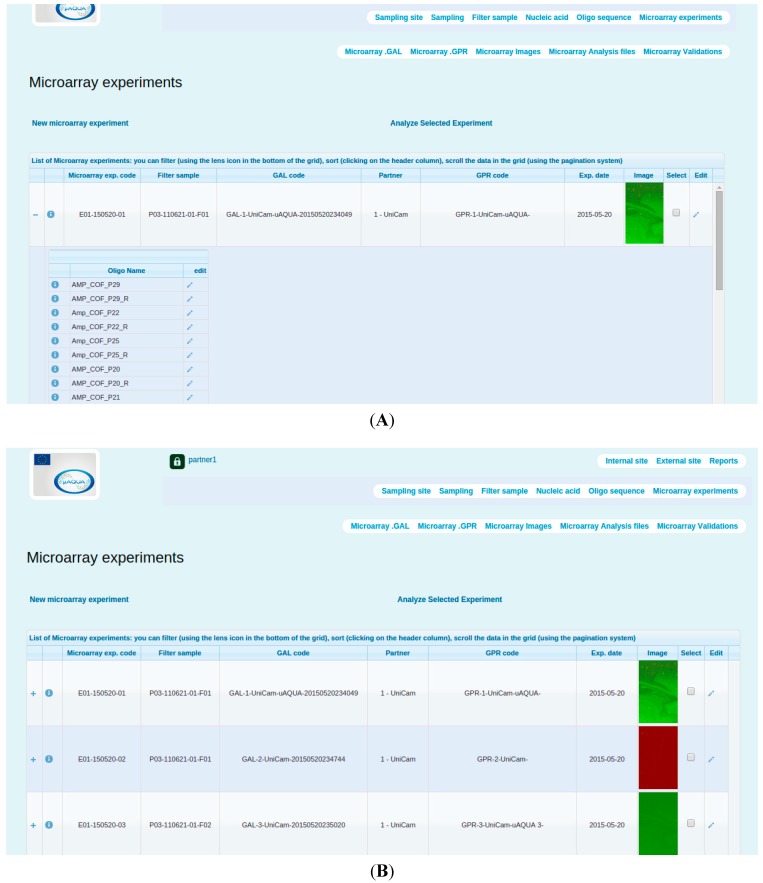
(**A**) An image showing *Microarray Experiments* module. The display includes a unique microarray experiment and a list of oligonucleotides used in this particular microarray experiment; (**B**) Display of saved microarray experiments.

SaDA summarizes multiple microarray experiments under *Microarray experiments* section (Figure 3). In this section, user can keep track of the sample utilized in each microarray experiment. The *Microarray experiment* section further contains other sections encapsulating data associated with GAL (array data) and GPR (hybridization data) files. Users can manually upload and download files, check the summary and perform downstream data analysis. 

In addition to the sampling and experimental modules, SaDA has other data containers for storing users and project partners’ related information. Access records are stored securely in encrypted form. SaDA provides access rights in three forms: view-only, access for editing and full edition and deletion rights.

### 2.4. Database Schema

SaDA currently consists of 53 MySQL relations covering different scientific procedure and different analytical data types. Structuring and managing database schema in Ruby on Rails is easy and efficient. 

### 2.5. Batch Uploads and Downloads of Data

To relieve researchers from the tedious task of entering data one by one, a set of batch file parsers were created. The upload of data files and images can be done asynchronously. The information generated during sampling procedures and oligonucleotides design can be uploaded in SaDA as plain comma-separated or tab-separated files. Files uploaded to SaDA are stored in a directory structure. The information within files is simultaneously stored in the database together with the meta-data for their quick and easy retrieval using SQL. Download of files is a straightforward approach via SaDA web interface. Download format is currently plain comma-separated or excel files.

## 3. Results and Discussion

In the present study we describe SaDA, a software to manage and analyze microarray-based datasets. There is no scarcity of freely available open source LIMS software solutions. Some of the LIMS-based solutions, for instance Base, iLAP, MARS, LabKey Server and PASSIM [22] are meaningful in big laboratories which, besides data analysis, are involved in protocol development, data acquisition and data sharing. Freely available tools for microarray analysis like TM4 [23], ArrayNorm [24], ArrayPipe [25] GPR-Analyzer [26] have either become obsolete or lack key features in terms of extensibility and integration with analysis tools, like R and modern graphing libraries. In the following sections we have described the key features of SaDA compared to any other light weighted domain specific LIMS. SaDA can be easily handled by a novice user. A working knowledge of system (Linux/UNIX/Mac OS/Microsoft Windows) tools installation is required to install, set up and use SaDA. However, if a user wants to extend SaDA’s functionalities and integrate new modules, knowledge of Ruby on Rails and R software programming is essential.

### 3.1. Utility of the SaDA Infrastructure

SaDA is a collection of well-integrated set of models with a very flexible and intuitive user interface: SaDA is developed to address the needs of many laboratories which produce their own batch of microarray data. The database backed software architecture aids in managing and representing oligonucleotide hybridization based microarray data. The flexible and generic database design facilitates mapping of the steadily changing laboratories work-flow. To allow the import of generic file formats, we have implemented a user definable parser that allows reading comma/tab delimited text files. Additionally, links to these files are maintained in a relational database to prevent the deletion of already imported, linked, or used files. The Microarray Gene Expression Data Society (MGED) develops and maintains standards for data acquisition, representation, and interchange such as the MIAME guidelines [27], the MAGE-TAB interchange format [28], and the MGED Ontology for microarray experiments [29]. SaDA does not enforce the use of the MGED standards. However it supports storage and retrieval of information required by MIAME. 

SaDA encapsulates open source software and graphing libraries: The usability of SaDA and the functionality of the interfaces are accounted by the inclusion of different models. These models encapsulate data from analytical pipelines of microarray analysis. To aid in fulfilling these functionalities, SaDA is supported by R programming software and jQWidgets [30] graphing libraries. SaDA utilizes RinRuby [31] gem to call R expressions/functions/script from Ruby on Rails interfaces. SaDA makes use of a jqGrid plugin [32] for a tabular data representation. 

SaDA facilitates comparative analysis of microarray results: SaDA implements an algorithm, running in the S2C server [33], for correlating microarray signal intensities with cell numbers from standard calibration plot to estimate cell counts. This way SaDA simplifies the prediction of cell counts from microarray data. With the implementation of flexible normalization procedures, it aids in the quantitative analysis and monitoring of microorganisms. In addition, the use of SaDA assists in making decisions entailing whether a cluster of species is below a set threshold and also in checking if poor hybridization quality or large proportion of nonspecific probe-targets interactions are responsible for negative results.

SaDA is extensible: Scientists have a natural inclination for software tools that can be customized to the particular needs of their labs. Tools for rapid modification and customization have proven particularly important in both the adoption of SaDA and the dissemination of MicroAQUA project. The concise and expressive nature of Ruby programming language provides API that is easy to change. There is a less boilerplate and a huge library of gems, including BioRuby project [34], designed for rails applications covering everything from integrating with networking tools to authorization products. The modular nature of Rails framework also supports integration of new features quickly without touching the application’s core components.

### 3.3. Limitations of SaDA

Most biomedical research applications are based on project specific guidelines. This limits the scope of any application to serve diverse purposes and creates boundaries of the platform that originates from its scientific approach. The main limitation encapsulating SaDA’s core functionality is its scientific focus. Currently it only implements microarray data management and analysis modules targeting Agilent’s single channel oligonucleotide hybridization technology. The current work does not aim to replace robust LIMS software solutions as it only targets a specific research study.

### 3.4. Availability and Future Directions

SaDA is open source software and is freely available as a GitHub repository for download from https://github.com/kumarsaurabh20/bioaqua1 under the terms of the Apache License 2.0 [35]. This site also provides documentation, tutorials and demos for users and developers, plus instructions for developers who wish to contribute code to the project through the SaDA GitHub repository. For its complete installation from scratch, we have developed a shell script which will install (for Linux based systems) all the components required for SaDA and clone SaDA automatically from the GitHub repositories. Since SaDA is a server application, it is platform independent and it can be run on any operating system. With some modifications, SaDA can also be made to run under other database environments. The other main requirement of SaDA is the working installation of R programming software. The shell script can detect the presence or absence of R software and in case it is not available on the system, the shell script will install it with the user permission. The future version will include handling and analysis of metagenomic data. The first implementation of SaDA was developed in the context of the EU FP7 project MicroAQUA which aimed at developing a universal microarray chip for the detection of fresh water pathogens. The data analysis steps in SaDA comply with the guidelines provided by MicroAQUA consortium.

There are many data-transformation and analysis algorithms that are feasible for integration in SaDA as an extended features and external modules. SaDA is developed keeping ecological studies and environmental monitoring in mind. Active development is underway to support other datasets which prove useful in this particular field of interest such as flow-cytometry datasets and their analytical representation, for the detection of toxins in the water based environmental systems. In addition, we are planning to extend the core functionality of SaDA to include metagenomic based data. Use of these standard technologies and datasets will allow interoperability of SaDA with other software. A major goal for SaDA development is the addition of support for other types of ecological high-throughput data. Development will be required to support the emergence of new arrays and platforms, particularly with respect to integrating results across different generations, and possibly different technologies. Support for experiments based on metagenomic data is the next immediate priority. These datasets enable high-throughput genome-scale investigations of interactions between genome and its surrounding environment. This extension will require the incorporation of new data analysis methods. An interface for combining microarray, metagenomics, proteomics and interaction based data within SaDA is already planned.

## 4. Conclusions

We have developed an integrated system consisting of a database that has been tailored to serve specific needs of microarray based environmental monitoring projects. Due to unique fusion of open source tools and libraries, the system can provide a model of building up a similar platform for other emerging genomics technologies. This utility enables users to collect, manipulate and analyze diverse and rapidly changing environmental data-set and their related biological data-types. The system allows easy customization of interfaces and visualization, thus providing a useful platform for collaborative research activities. Implementation of SaDA by the members of MicroAQUA consortium over a period of about three years where water sampling data, molecular analysis and high-throughput microarray experiments have been carried out, has helped to fine-tune the features of this software to suit the needs of a wide range of researchers. The successful application of SaDA suggests the platform has the potential to become an appropriate tool for a wide range of users focused not only in water based environmental research but also in other studies aimed at exploring and analyzing complex ecological habitats. 

## Supplementary Materials

Graphical workflows for SaDA’s core modules are available as supplementary materials and can be accessed at: http://www.mdpi.com/1660-4601/12/6/6352/.
