# Supplementary material for: SaDA: From Sampling to Data Analysis—An Extensible Open Source Infrastructure for Rapid, Robust and Automated Management and Analysis of Modern Ecological High-Throughput Microarray Data
Source: Int J Environ Res Public Health. 2015 Jun 3;12(6):6352–66. doi: 10.3390/ijerph120606352 (PMC4483705; doi:10.3390/ijerph120606352)
Supplement: Supplementary file 1 [file ijerph-12-06352-s001.zip › 1_Login.pdf]

General Information about the MicroAqua internal website

Login

Reach the Login page by clicking “Internal Site” and sign in with your credentials

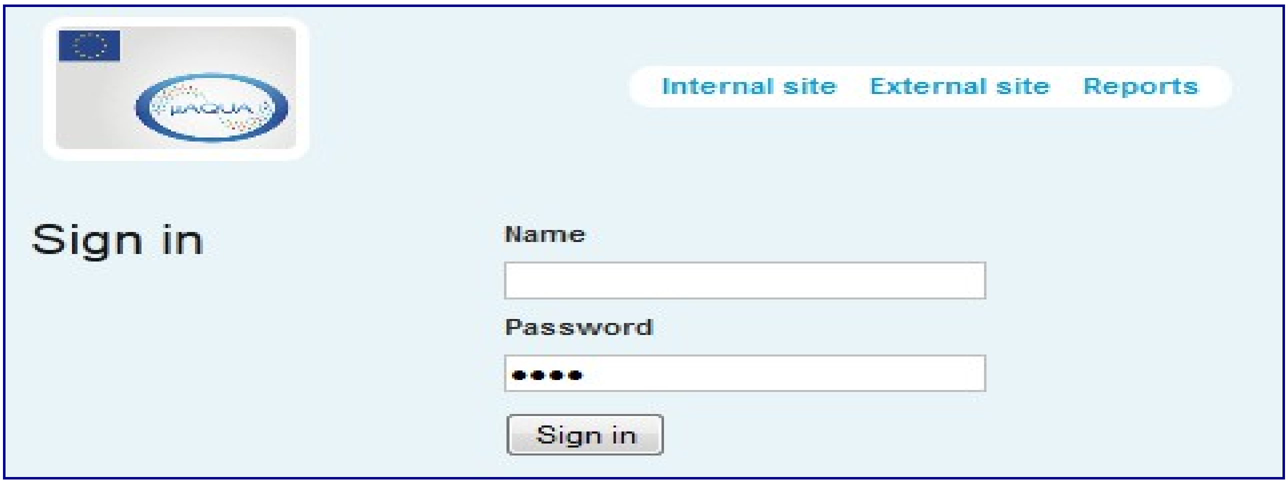

Internal site

The site covers three major areas:

Samples
Filter Samples
Extracted Nucleic acids(RNA/DNA)
Oligo sequences
Microarray

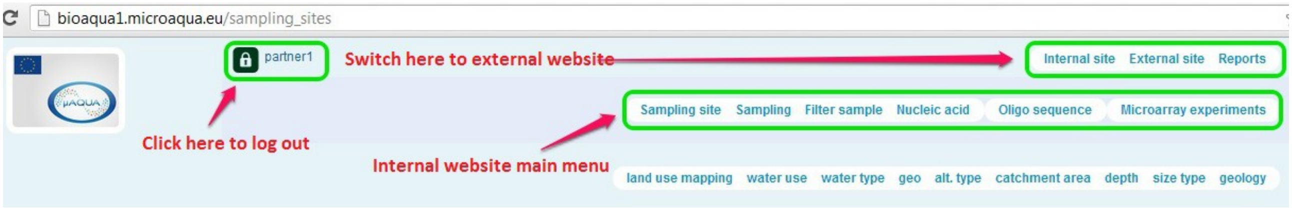

Once logged it appears the main menu. In the top right you can reach the External web site or the Reports site.

Architecture

The Internal Site is separated from the External Site. They are developed with different technologies as their scope is different.

External site (Web content) is powered by Drupal (CMS).

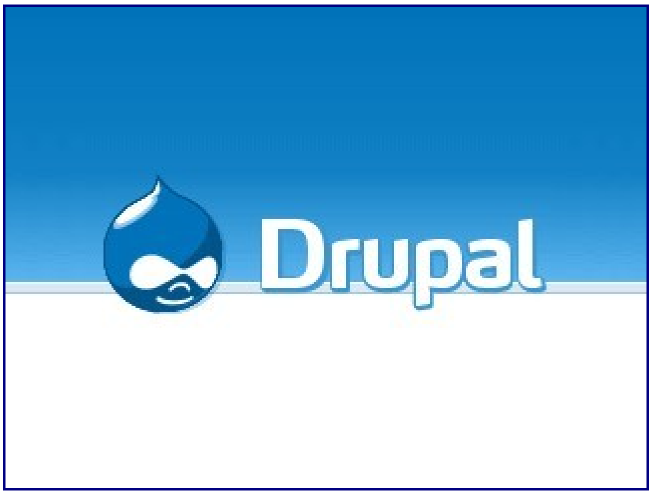

Internal site (Database) is developed under Ruby on Rails (MVC).

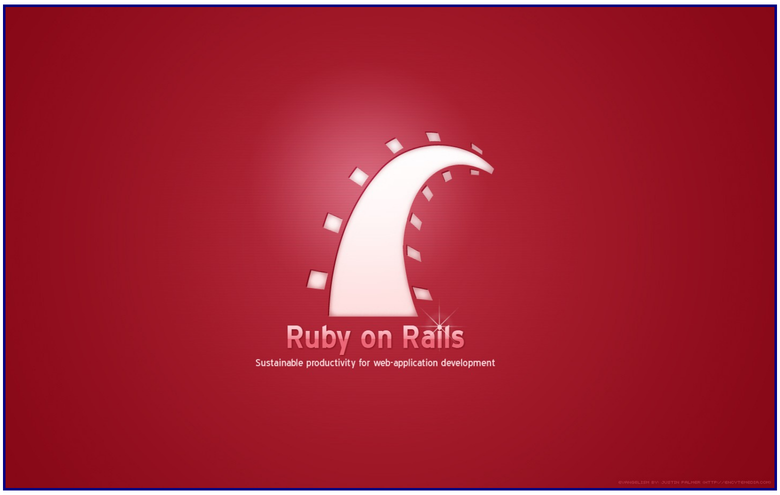

For this reason the authentication systems are separated. The default password is the same in both systems, but you are free to change the passwords and keep them synchronized.
