# Supplementary material for: SaDA: From Sampling to Data Analysis—An Extensible Open Source Infrastructure for Rapid, Robust and Automated Management and Analysis of Modern Ecological High-Throughput Microarray Data
Source: Int J Environ Res Public Health. 2015 Jun 3;12(6):6352–66. doi: 10.3390/ijerph120606352 (PMC4483705; doi:10.3390/ijerph120606352)

Sampling Sites

This section lists all the inserted sampling sites.

The values for the attributes of a sampling can be personalized using the contextual menu.

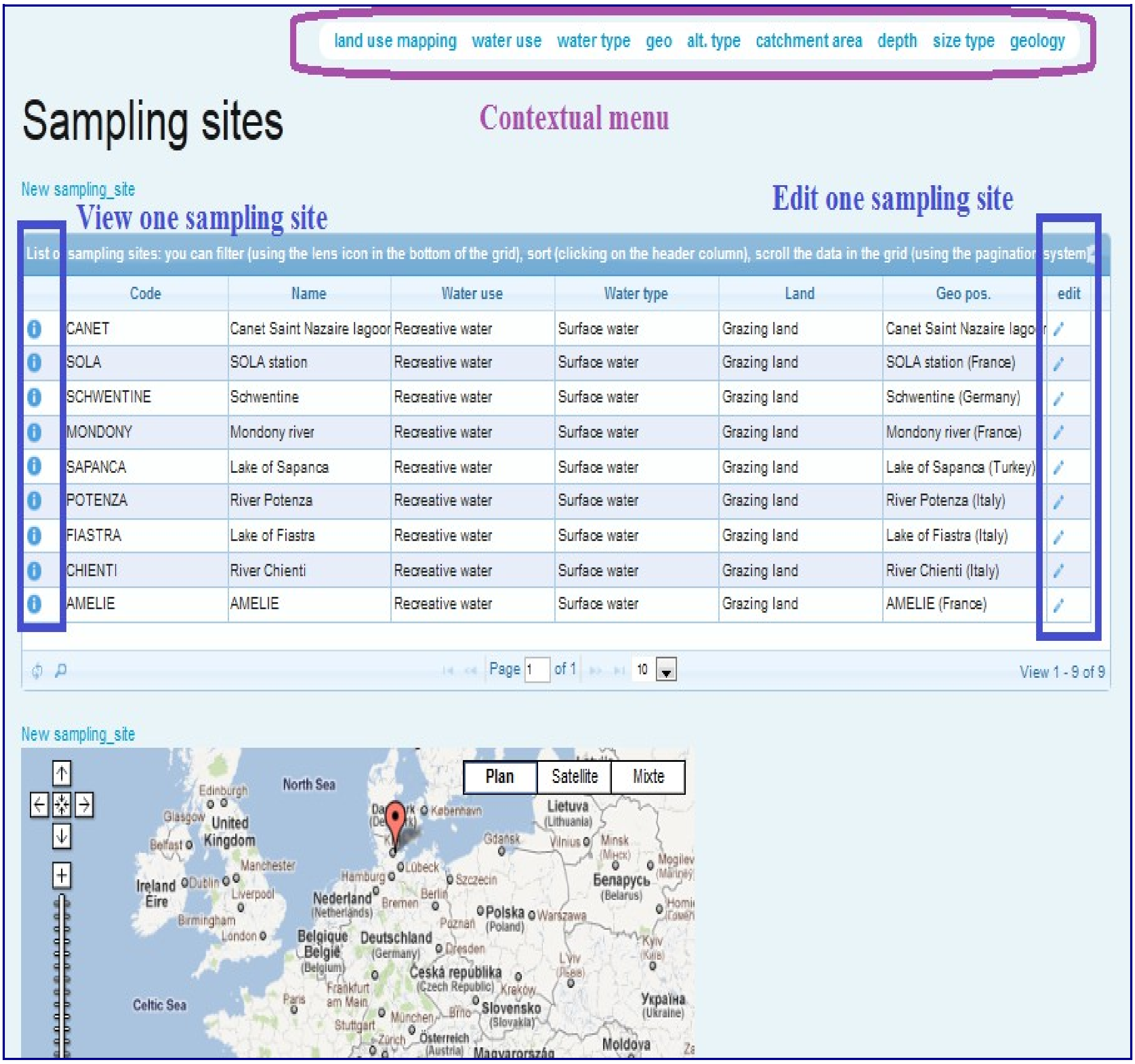

To create a new sampling site click on the link "New sampling site"

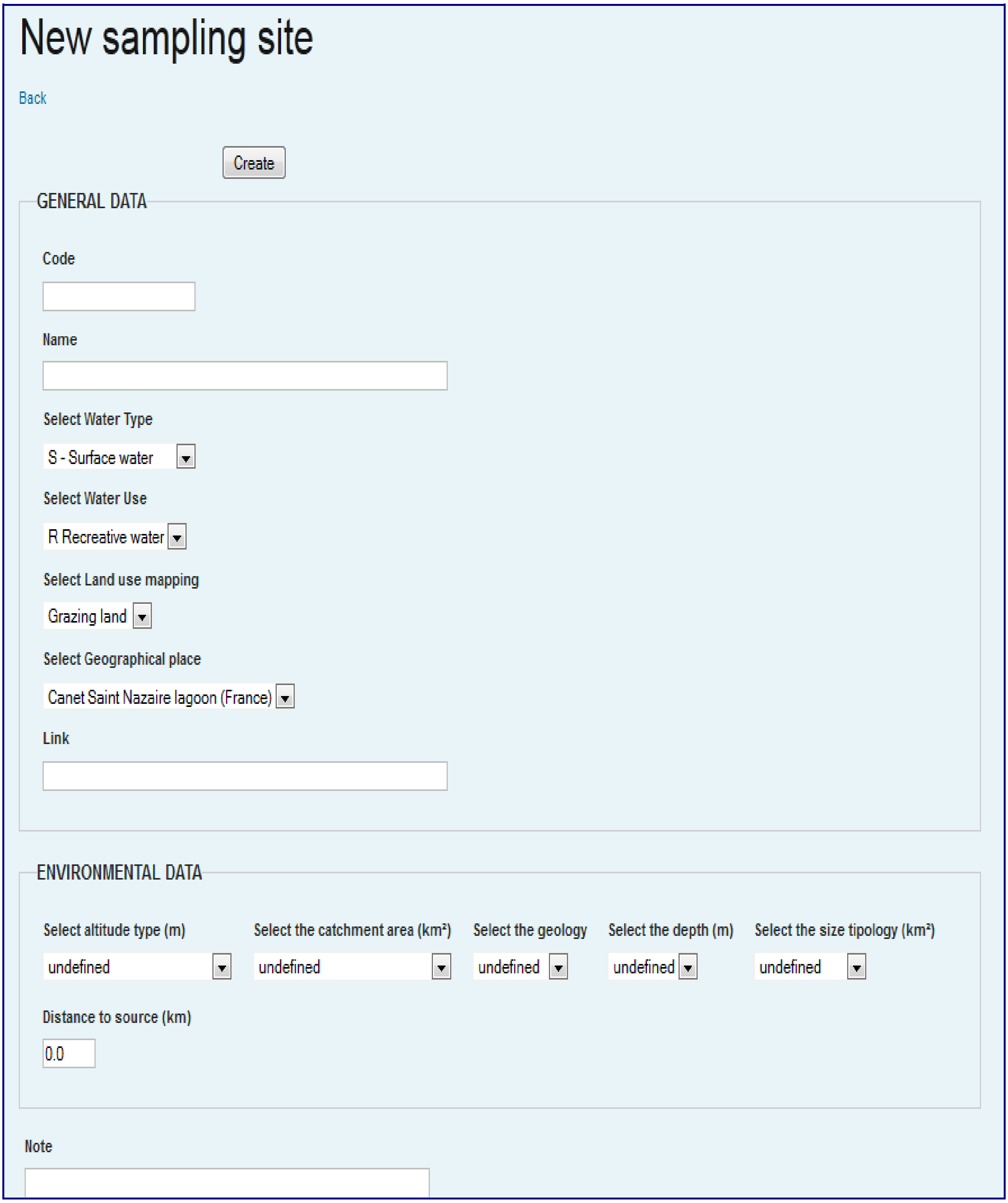

Clicking on one link in the contextual menu of a main scope (Samplings, Oligo and MicroArray) brings to the relative resource (e.g. water use or water type).

Usually the resource appears like a drop-down box where you can select a choice.

The list of available values for the resource can be extended using the dedicated page.

All resources have these pages:

1. Page for listing the elements
2. Page to create a new element
3. Page to modify an existing element.

For example the geographical position ("geo") resource.

land use mapping | water use | water type | geo | alt. type | catchment area | depth | size type | geology | country

Geographical positions

New geo

Name	Lon	Lat	Country			
Canet Saint Nazaire lagoon	3.029523	42.659567	FR France	Show	Edit	Delete
SOLA station	3.029523	42.659567	FR France	Show	Edit	Delete
Schwentine	10.18611	54.3275	DE Germany	Show	Edit	Delete
Mondony river	2.669206	42.472034	FR France	Show	Edit	Delete
Lake of Sapanca	30.25	40.716667	TR Turkey	Show	Edit	Delete
River Potenza	12.92254	43.130174	IT Italy	Show	Edit	Delete
Lake of Fiastra	13.1562	43.0359	IT Italy	Show	Edit	Delete
River Chienti	13.033333	42.95	IT Italy	Show	Edit	Delete
AMELIE	3.029523	42.659567	FR France	Show	Edit	Delete

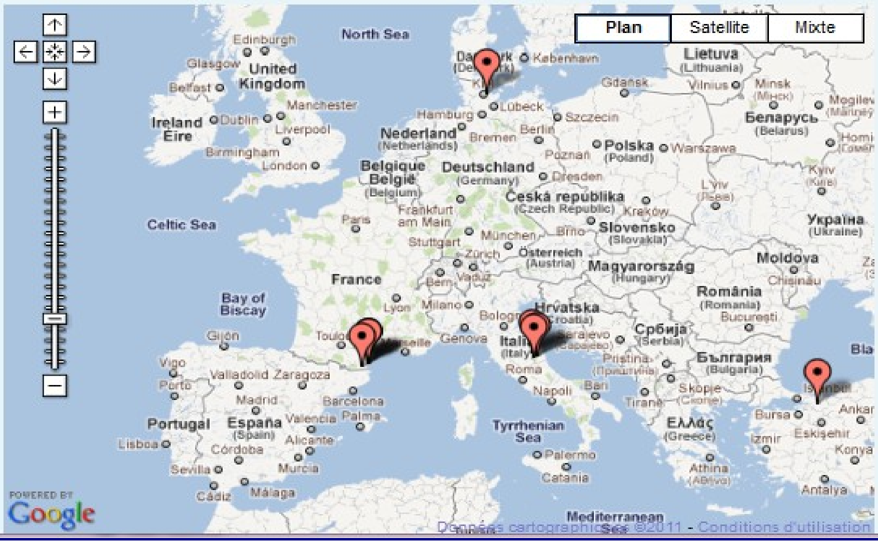

Supplement: Supplementary file 1 [file ijerph-12-06352-s001.zip › 2_Sampling_sites.pdf]
