# Supplementary material for: SaDA: From Sampling to Data Analysis—An Extensible Open Source Infrastructure for Rapid, Robust and Automated Management and Analysis of Modern Ecological High-Throughput Microarray Data
Source: Int J Environ Res Public Health. 2015 Jun 3;12(6):6352–66. doi: 10.3390/ijerph120606352 (PMC4483705; doi:10.3390/ijerph120606352)
Supplement: Supplementary file 1 [file ijerph-12-06352-s001.zip › 3_Samplings.pdf]

Samplings

Each water sample is linked to one sampling site and can have several filter samples (visible clicking on the + on the sample).

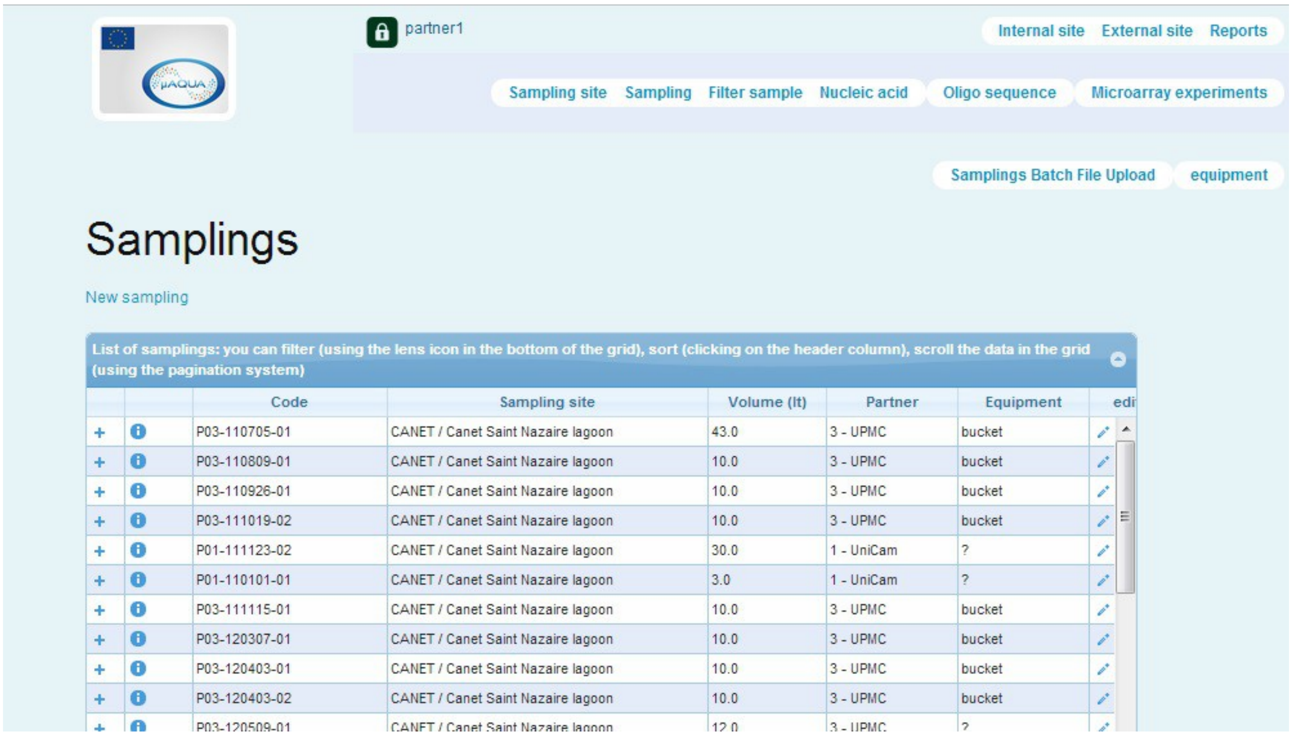

Click on the link "*New sampling*" to access the creation page

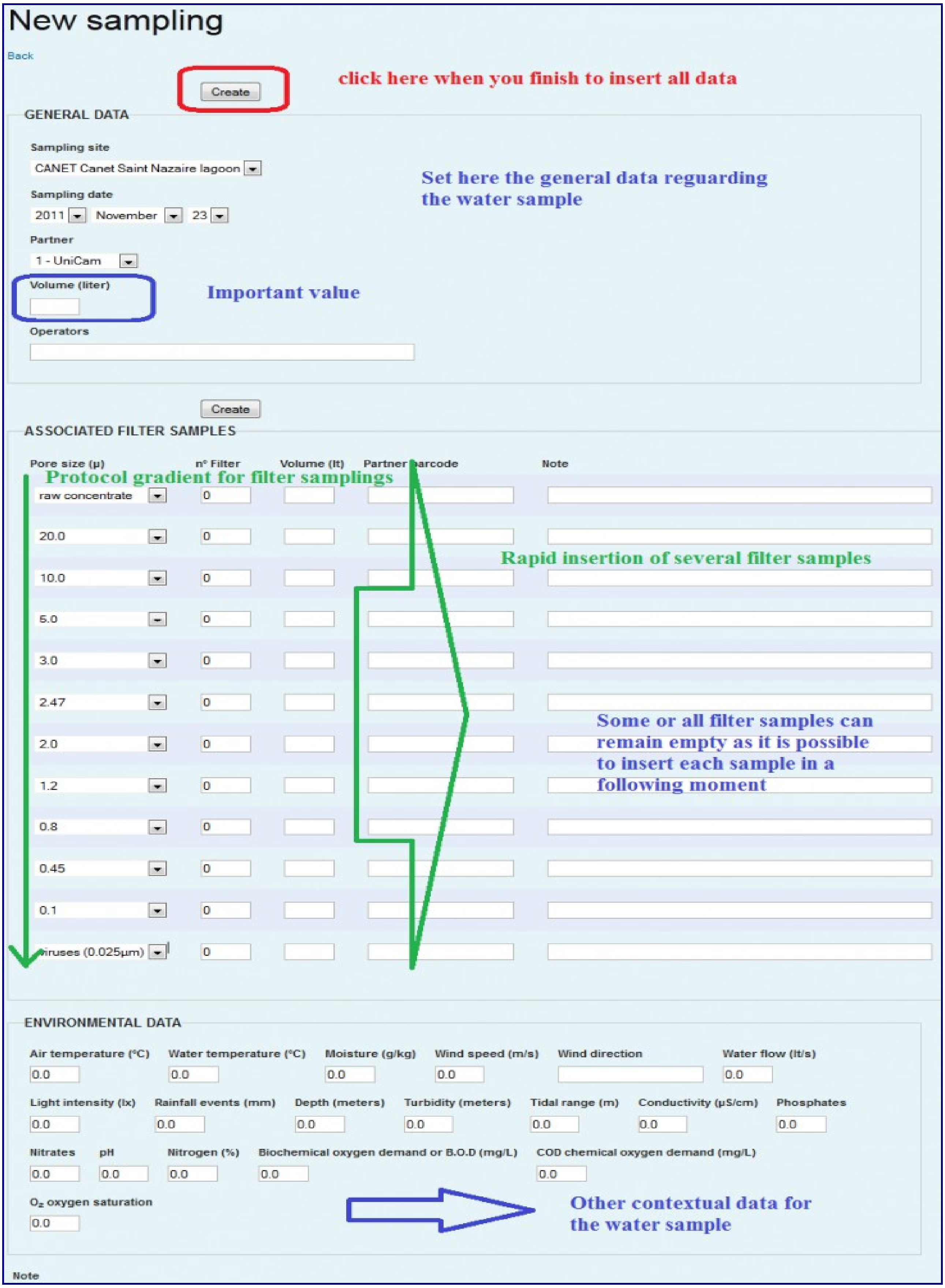
New sampling
Back
Create
click here when you finish to insert all data
GENERAL DATA
Sampling site
CANET Canet Saint Nazaire lagoon
Set here the general data reguarding the water sample
Sampling date
2011 November 23
Partner
1 - UniCam
Volume (liter)
Important value
Operators
Create
ASSOCIATED FILTER SAMPLES
Pore size (µ)
n° Filter
Volume (lt)
Partner barcode
Note
Protocol gradient for filter samplings
raw concentrate
20.0
10.0
5.0
3.0
2.47
2.0
1.2
0.8
0.45
0.1
viruses (0.025µm)
Rapid insertion of several filter samples
Some or all filter samples can remain empty as it is possible to insert each sample in a following moment
ENVIRONMENTAL DATA
Air temperature (°C)
Water temperature (°C)
Moisture (g/kg)
Wind speed (m/s)
Wind direction
Water flow (lt/s)
Light intensity (lx)
Rainfall events (mm)
Depth (meters)
Turbidity (meters)
Tidal range (m)
Conductivity (µS/cm)
Phosphates
Nitrates
pH
Nitrogen (%)
Biochemical oxygen demand or B.O.D (mg/L)
COD chemical oxygen demand (mg/L)
O2 oxygen saturation
Other contextual data for the water sample
Note

Filter Sample

A partner customized barcode can be associated to each filter sample.

As for the samplings the system generates automatically a MicroAqua code.

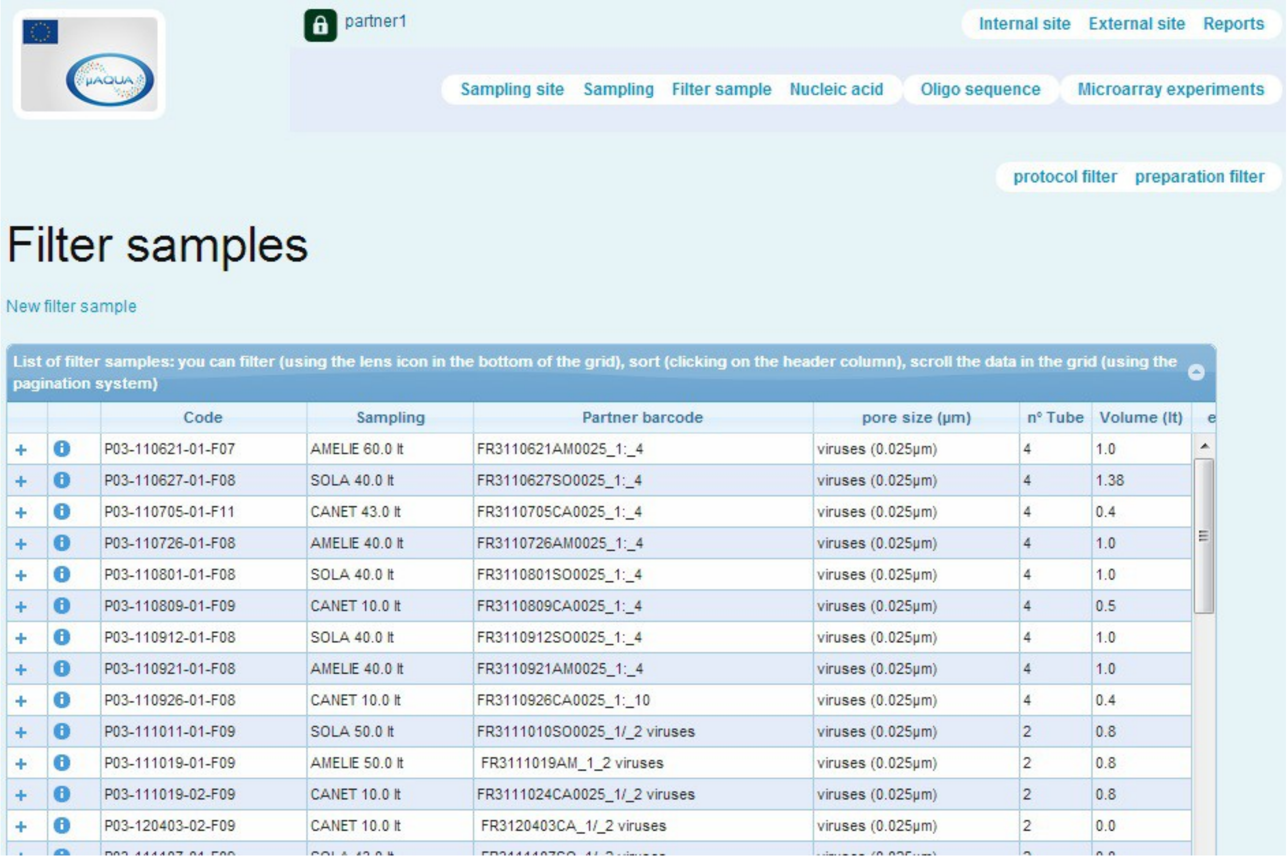

		Code	Sampling	Partner barcode	pore size (µm)	n° Tube	Volume (lt)
+	i	P03-110621-01-F07	AMELIE 60.0 lt	FR3110621AM0025_1:_4	viruses (0.025µm)	4	1.0
+	i	P03-110627-01-F08	SOLA 40.0 lt	FR3110627SO0025_1:_4	viruses (0.025µm)	4	1.38
+	i	P03-110705-01-F11	CANET 43.0 lt	FR3110705CA0025_1:_4	viruses (0.025µm)	4	0.4
+	i	P03-110726-01-F08	AMELIE 40.0 lt	FR3110726AM0025_1:_4	viruses (0.025µm)	4	1.0
+	i	P03-110801-01-F08	SOLA 40.0 lt	FR3110801SO0025_1:_4	viruses (0.025µm)	4	1.0
+	i	P03-110809-01-F09	CANET 10.0 lt	FR3110809CA0025_1:_4	viruses (0.025µm)	4	0.5
+	i	P03-110912-01-F08	SOLA 40.0 lt	FR3110912SO0025_1:_4	viruses (0.025µm)	4	1.0
+	i	P03-110921-01-F08	AMELIE 40.0 lt	FR3110921AM0025_1:_4	viruses (0.025µm)	4	1.0
+	i	P03-110926-01-F08	CANET 10.0 lt	FR3110926CA0025_1:_10	viruses (0.025µm)	4	0.4
+	i	P03-111011-01-F09	SOLA 50.0 lt	FR3111010SO0025_1/_2 viruses	viruses (0.025µm)	2	0.8
+	i	P03-111019-01-F09	AMELIE 50.0 lt	FR3111019AM_1_2 viruses	viruses (0.025µm)	2	0.8
+	i	P03-111019-02-F09	CANET 10.0 lt	FR3111024CA0025_1/_2 viruses	viruses (0.025µm)	2	0.8
+	i	P03-120403-02-F09	CANET 10.0 lt	FR3120403CA_1/_2 viruses	viruses (0.025µm)	2	0.0

Clicking on "*New filter sample*" it is possible to reach the page for creating a single filter sample (to be associated to a water sample).
