# Supplementary material for: SaDA: From Sampling to Data Analysis—An Extensible Open Source Infrastructure for Rapid, Robust and Automated Management and Analysis of Modern Ecological High-Throughput Microarray Data
Source: Int J Environ Res Public Health. 2015 Jun 3;12(6):6352–66. doi: 10.3390/ijerph120606352 (PMC4483705; doi:10.3390/ijerph120606352)
Supplement: Supplementary file 1 [file ijerph-12-06352-s001.zip › 4_Taxonomy_browser.pdf]

Taxonomy Browser

For MicroAQUA, probes have been designed for taxonomic groups like class level, clade level, genus and species level. The hierarchial probes make it easier to analyze environmental samples. For the species level probe to be present, the entire taxonomic hierarchy above the species probe has to be present. If the taxomomic hierarchy shows no signal, then the signal from the species probe can be considered to be a false positive.

To assign probes, saved in database, a "Taxonomy browser" is implemented on the MicroAqua internal website.

The following instruction will explain how to make use of the taxonomy browser tool :

1. To access the browser, click on the edit button, the last column of the grid, of individual row/probe.

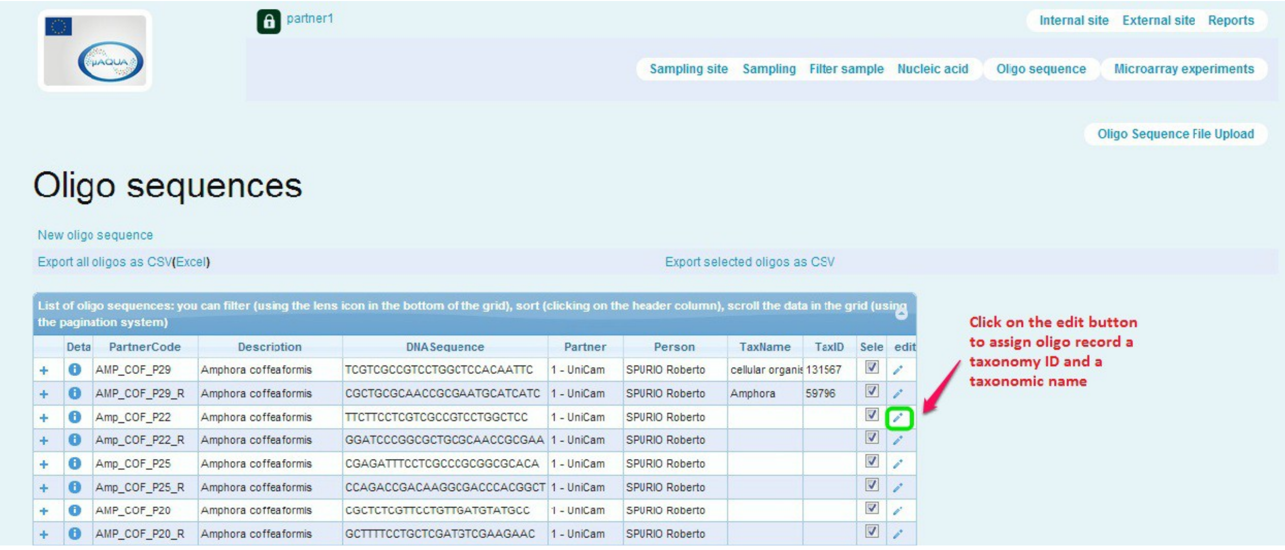

2. Scroll down to the "Organism" field set, and either enter a name of the species against which the oligo was designed or, if available, enter the NCBI taxomic ID and click "Find"

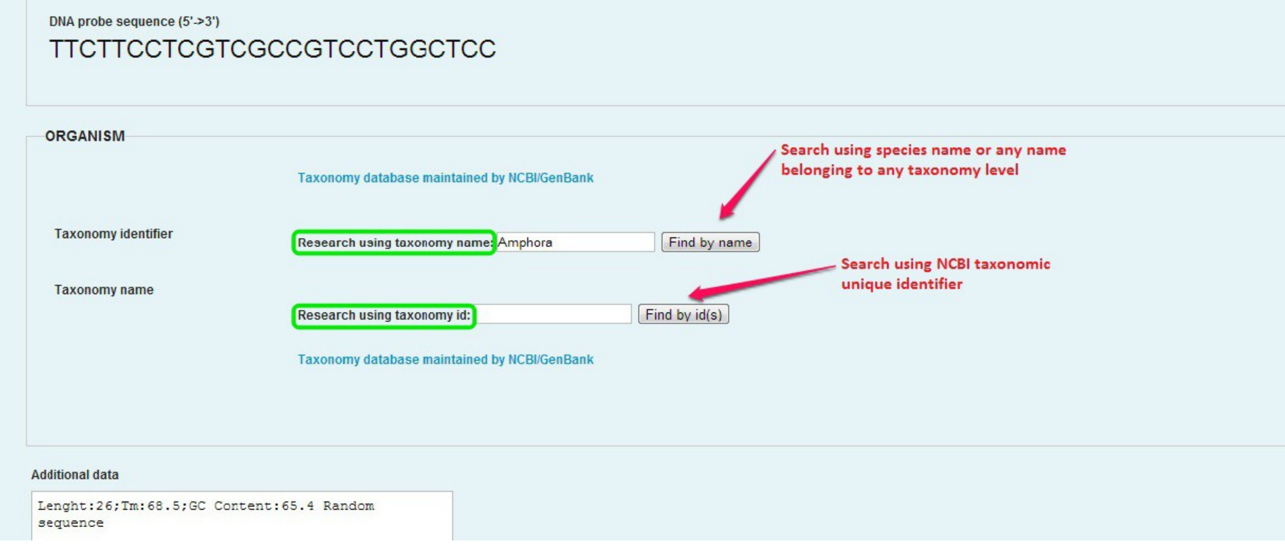

3. The browser will display a complete classification of the species and all the levels available for the particular species in NCBI database. To select any level and ID, simply click on the button, available in front of names in green.

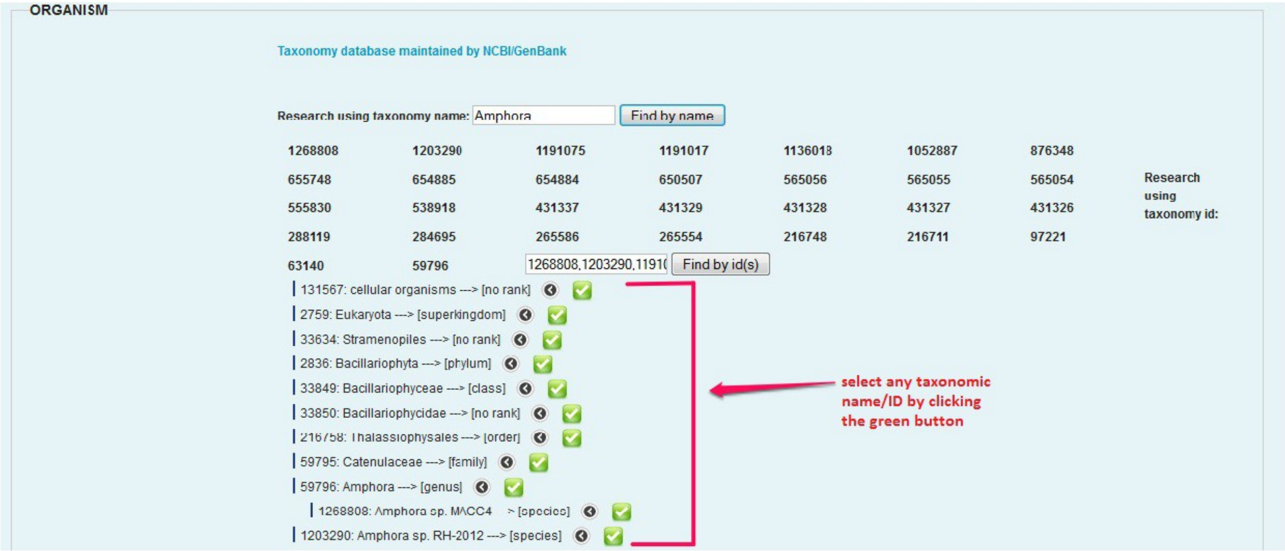

4. The selected oligo will automatically get updated with the taxonomy name and ID.

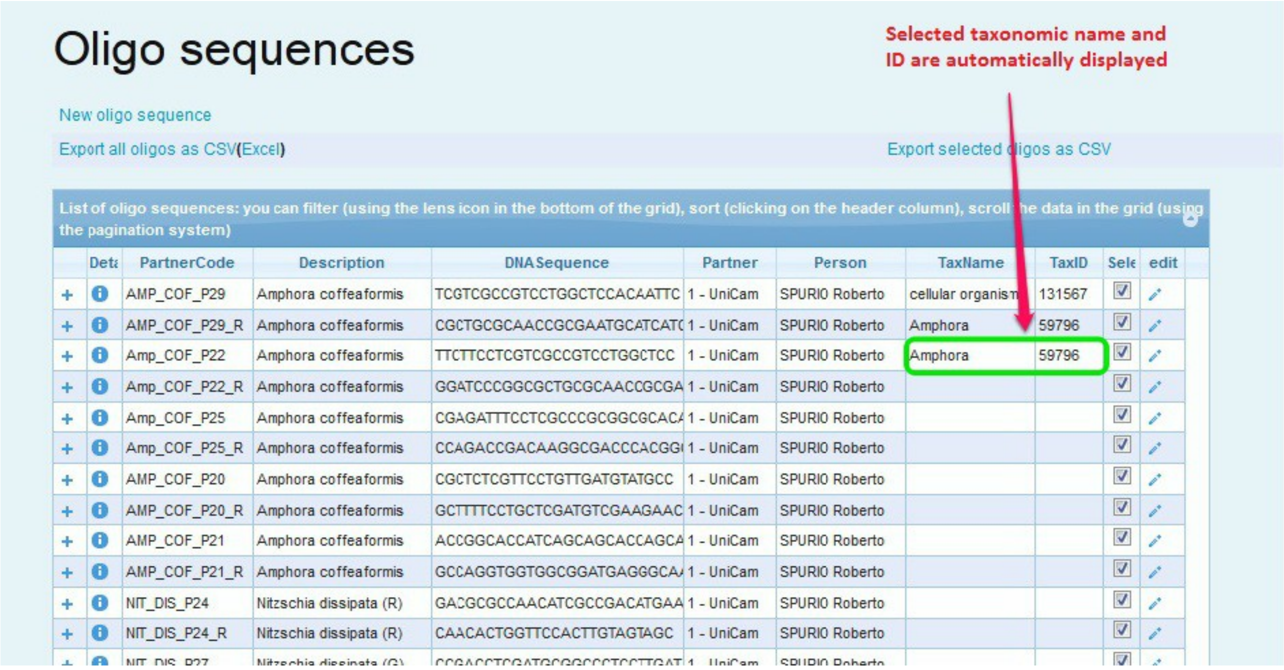

Deta	PartnerCode	Description	DNASequence	Partner	Person	TaxName	TaxID	Sele	edit
	AMP_COF_P29	Amphora coffeaformis	TCGTCGCCGTCCTGGCTCCACAATTC	1 - UniCam	SPURIO Roberto	cellular organism	131567		
	AMP_COF_P29_R	Amphora coffeaformis	CGCTGCGCAACCGCGAATGCATCAT	1 - UniCam	SPURIO Roberto	Amphora	59796		
	Amp_COF_P22	Amphora coffeaformis	TTCTTCCTCGTCGCCGTCCTGGCTCC	1 - UniCam	SPURIO Roberto	Amphora	59796		
	Amp_COF_P22_R	Amphora coffeaformis	GGATCCCGGCGCTGCGCAACCGCGA	1 - UniCam	SPURIO Roberto				
	Amp_COF_P25	Amphora coffeaformis	CGAGATTTCCTCGCCCGCGGCGCAC	1 - UniCam	SPURIO Roberto				
	Amp_COF_P25_R	Amphora coffeaformis	CCAGACCGACAAGGCGACCCACGG	1 - UniCam	SPURIO Roberto				
	AMP_COF_P20	Amphora coffeaformis	CGCTCTCGTTCCTGTTGATGTATGCC	1 - UniCam	SPURIO Roberto				
	AMP_COF_P20_R	Amphora coffeaformis	GCTTTTCCTGCTCGATGTCGAAGAAC	1 - UniCam	SPURIO Roberto				
	AMP_COF_P21	Amphora coffeaformis	ACCGGCACCATCAGCAGCACCAGCA	1 - UniCam	SPURIO Roberto				
	AMP_COF_P21_R	Amphora coffeaformis	GCCAGGTGGTGGCGGATGAGGGCA	1 - UniCam	SPURIO Roberto				
	NIT_DIS_P24	Nitzschia dissipata (R)	GACGCGCCAACATCGCCGACATGAA	1 - UniCam	SPURIO Roberto				
	NIT_DIS_P24_R	Nitzschia dissipata (R)	CAACACTGGTTCCACTTGTAGTAGC	1 - UniCam	SPURIO Roberto				
