# Supplementary material for: SaDA: From Sampling to Data Analysis—An Extensible Open Source Infrastructure for Rapid, Robust and Automated Management and Analysis of Modern Ecological High-Throughput Microarray Data
Source: Int J Environ Res Public Health. 2015 Jun 3;12(6):6352–66. doi: 10.3390/ijerph120606352 (PMC4483705; doi:10.3390/ijerph120606352)

Batch upload of data files

Samplings
Oligo Sequence

Batch upload of data files is implemented on the MicroAqua internal website to ease uploading and parsing large chunks of data. Currently this functionality is available for "Sampling" and "Oligo sequence" data.

1. Click on the button for "sampling batch file uplaod", under Samplings section.

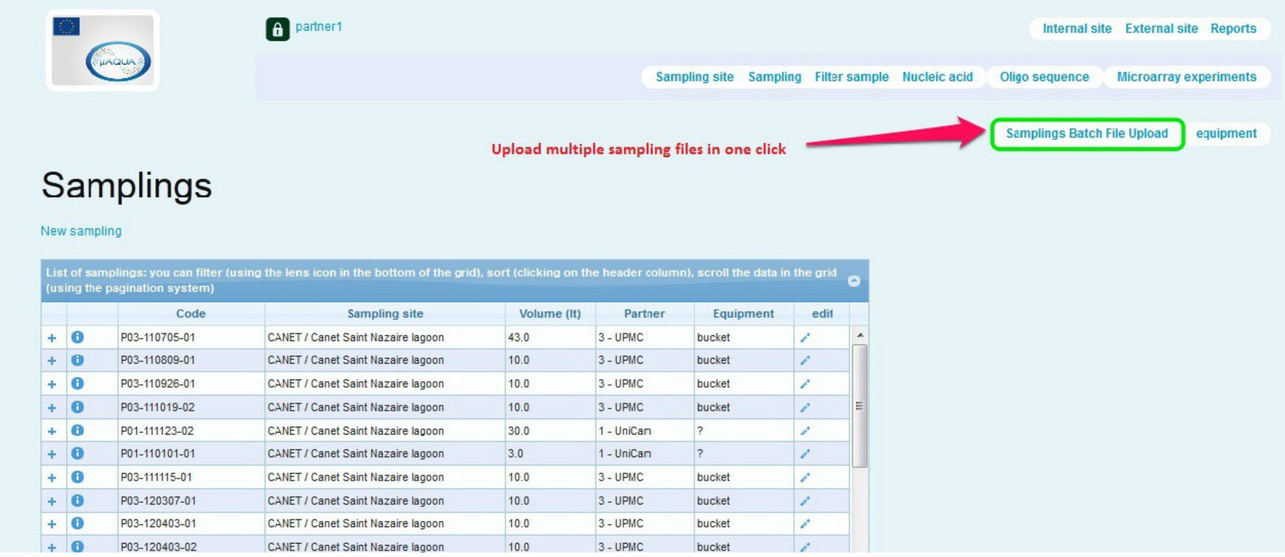

2. The data file can be uploaded in formats like .csv(comma separated values) or .tsv(tab separated values). A template for the sample data file is available at the link "Download an example of sampling file". Just download it and fill it with your data.

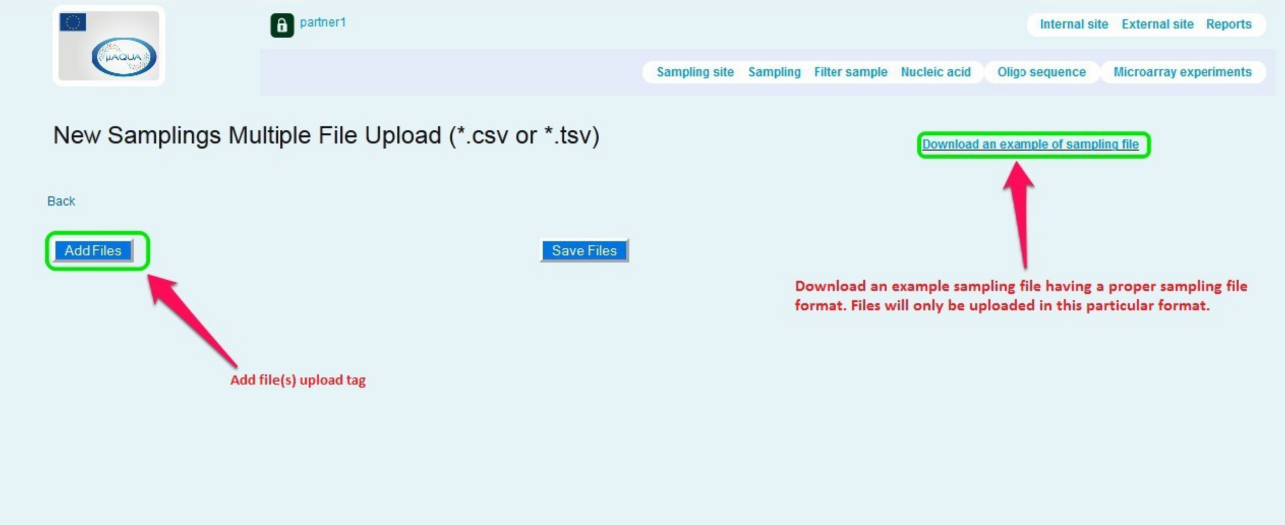

3. To fill data in the example file, some rules have to be taken care of:

a) In the first field, Sampling Site, fill the blank field by Sampling Site *Code*. Codes are available in the index table of *MicroAqua internal website > Sampling Site*.

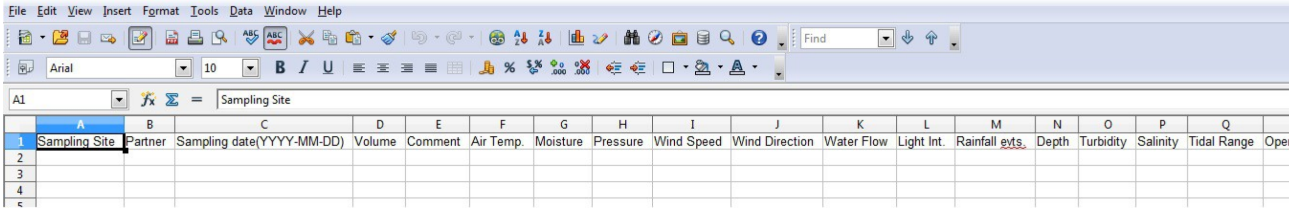

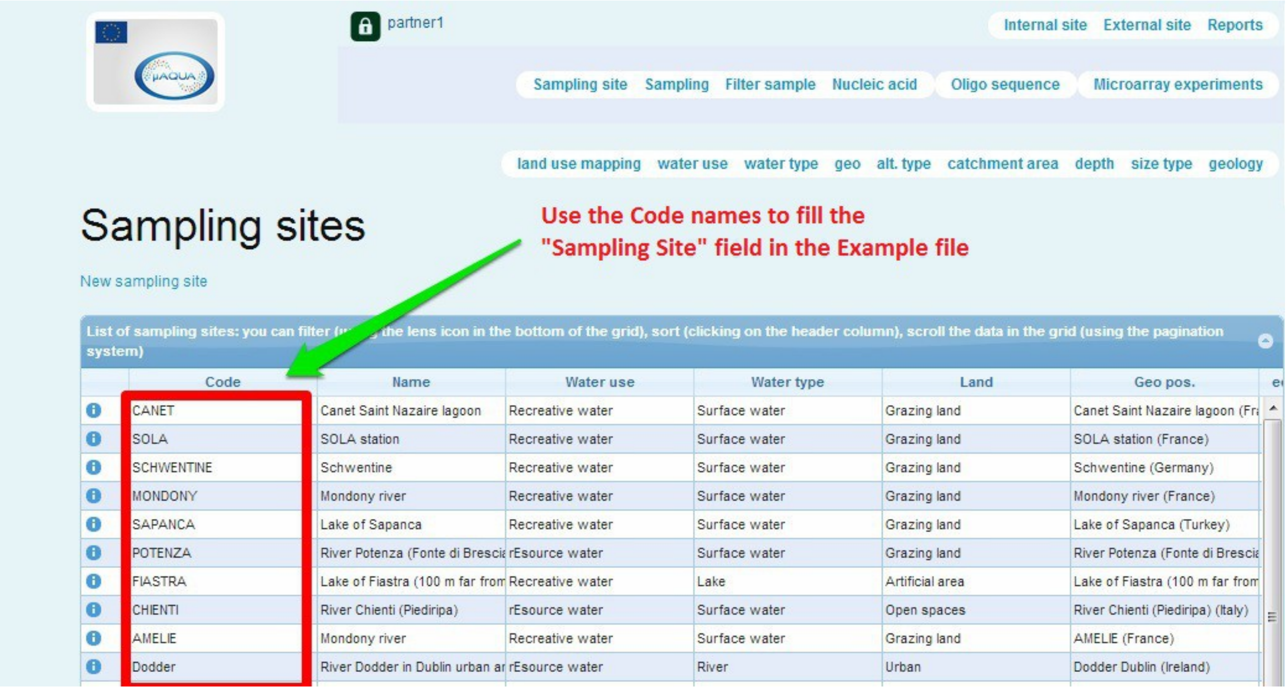

Code	Name	Water use	Water type	Land	Geo pos.
CANET	Canet Saint Nazaire lagoon	Recreative water	Surface water	Grazing land	Canet Saint Nazaire lagoon (Fr
SOLA	SOLA station	Recreative water	Surface water	Grazing land	SOLA station (France)
SCHWENTINE	Schwentine	Recreative water	Surface water	Grazing land	Schwentine (Germany)
MONDONY	Mondony river	Recreative water	Surface water	Grazing land	Mondony river (France)
SAPANCA	Lake of Sapanca	Recreative water	Surface water	Grazing land	Lake of Sapanca (Turkey)
POTENZA	River Potenza (Fonte di Brescia	rEsource water	Surface water	Grazing land	River Potenza (Fonte di Brescia
FIASTRA	Lake of Fiastra (100 m far from	Recreative water	Lake	Artificial area	Lake of Fiastra (100 m far from
CHIENTI	River Chienti (Piedripa)	rEsource water	Surface water	Open spaces	River Chienti (Piedripa) (Italy)
AMELIE	Mondony river	Recreative water	Surface water	Grazing land	AMELIE (France)
Dodder	River Dodder in Dublin urban ar	rEsource water	River	Urban	Dodder Dublin (Ireland)

b) For Partners field, put the Partners code (in upper or lower case). Refer to the following list or check the codes from the MicroAqua website *Partners* page (http://www.microaqua.eu/beneficiaries).

University of Camerino	**UNICAM**
University of Portsmouth	**UniPort**
Universite Pierre et Marie Curie	**UPMC**
Veolia Environment Recherche et innovation	**VERI**
Istituto Superiore Di Sanita	**ISS**
University of Ireland, Dublin	**UCD**
National Center of Infectious and Parasitic Diseases	**NCIPD**
Queens University Belfast	**QUB**
MariLim Gesellschaft fur Gewasseruntersuchung mbH	**Marilim**
Istanbul University	**UniIst**
Universidade de Santiago de Compostela	**USC**
Scienion AG, Berlin	**SCIE**

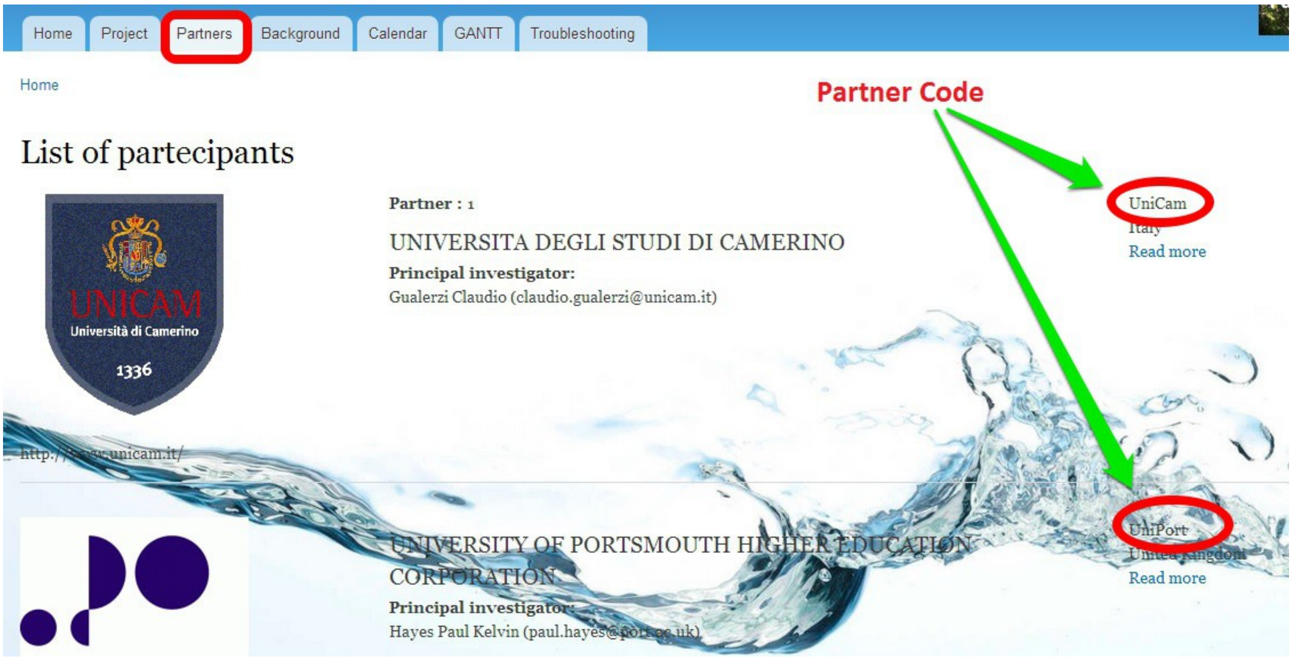

Save the Example file with the name of your choice and now its ready to be uploaded.

NOTE1: The file is already in .CSV format. There is no need to change the format of the file.

NOTE2: It is essential to fill in all the field available in the Example file. If the Partner has no data for the respective field, especially for Enviornmental data like pressure, moisture etc, just put "0.0" value in the field. Do not leave any field blank.

4. Once the data files are ready, simply click on "Add files" button *multiple times* to get the file upload tag. Maximum of 10 files can be uploaded together with one click. Just browse your files and provide the location. Then click on "Save Files". A unique sampling code will be assigned to individual rows with in the data file. Once the data is parsed sucessfully, filter samples can be associated manually with each sampling entry.

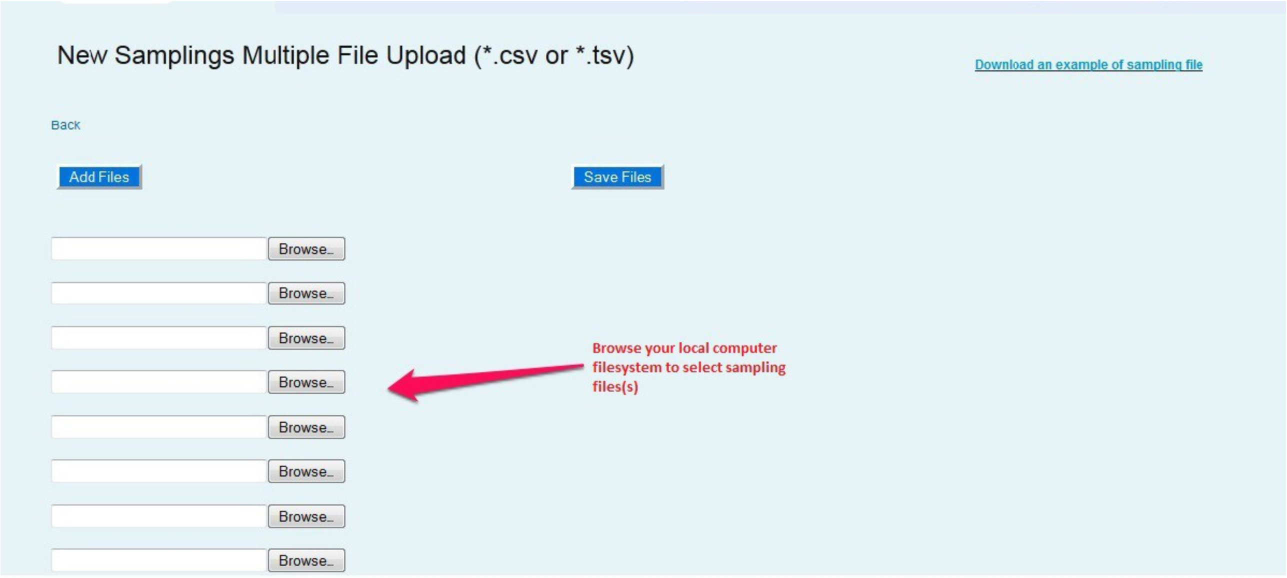

5. The same procedure has to be followed with "Oligo Sequence" batch files.

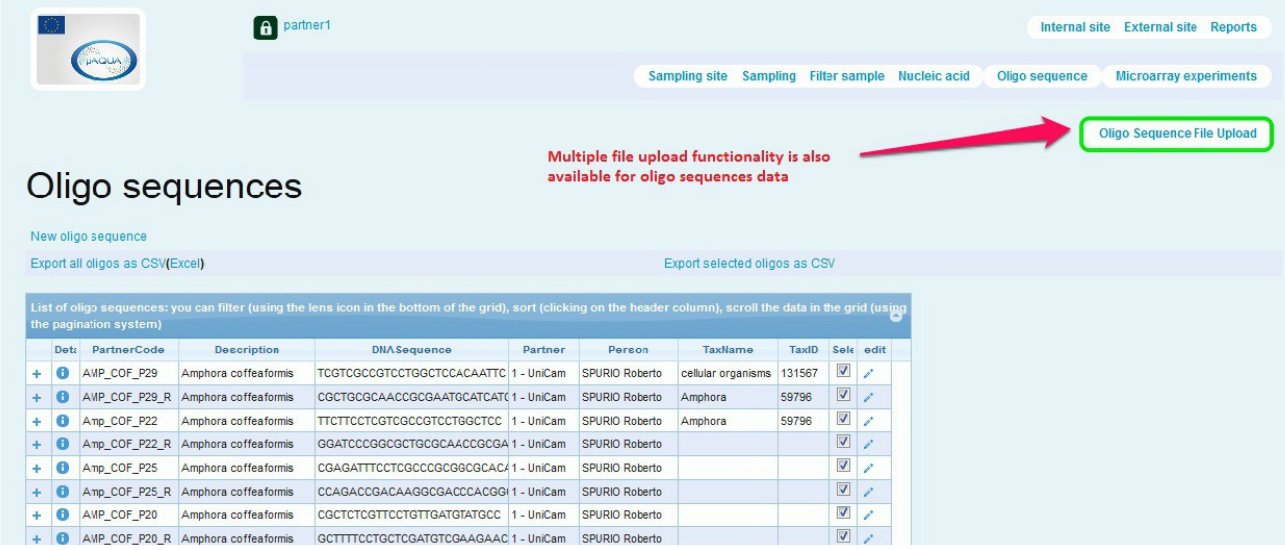

6. In the example file, fill the *Partner* field with partner code and *Person* field with the Firstnames of the person uploading the file. For partner please refer to the section **3.b** above and for Persons, please refer the following image. Firstnames of all the persons involved in MicroAqua are available here: http://bioaqua1.microaqua.eu/people

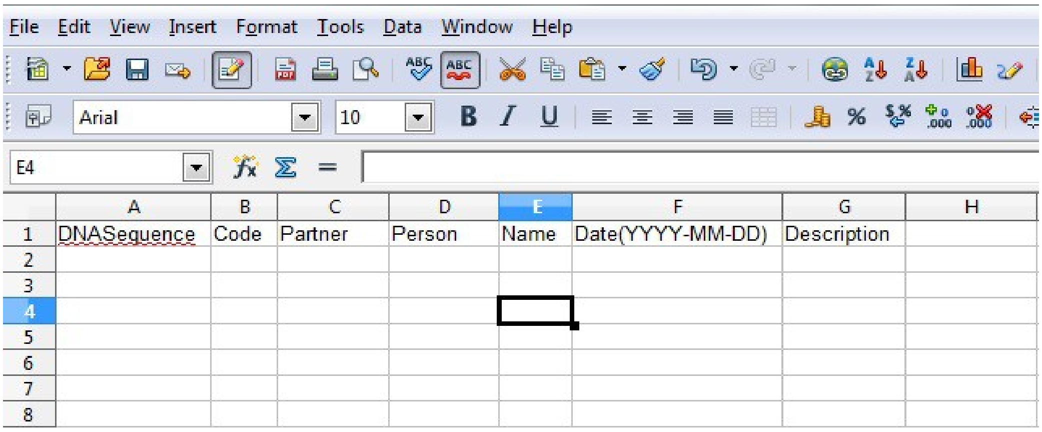

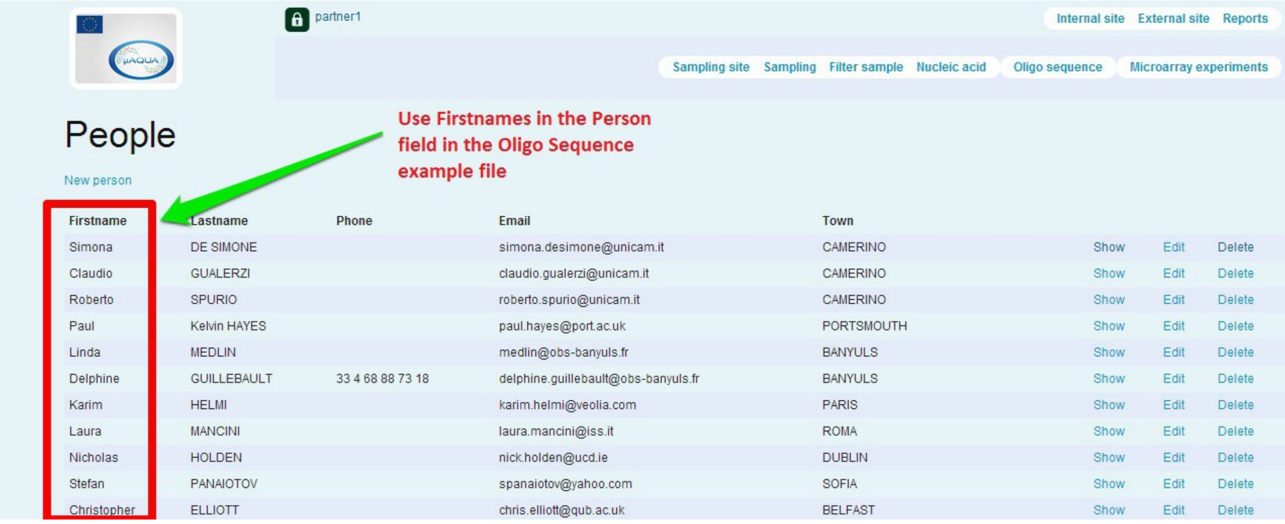

Supplement: Supplementary file 1 [file ijerph-12-06352-s001.zip › 5_Batch_upload.pdf]
