# Supplementary material for: SaDA: From Sampling to Data Analysis—An Extensible Open Source Infrastructure for Rapid, Robust and Automated Management and Analysis of Modern Ecological High-Throughput Microarray Data
Source: Int J Environ Res Public Health. 2015 Jun 3;12(6):6352–66. doi: 10.3390/ijerph120606352 (PMC4483705; doi:10.3390/ijerph120606352)

<u>Microarray Experiments</u>

Microarray GAL
Microarray GPR
Microarray Images

The current implementation of data management support in the MicroAqua website includes .gal, .gpr & image file upload and parsing of file associated data to populate the current MicroAQUA database for better visualization, handling and further analysis of Microarray data.

Following are the steps to operate the utility on MicroAqua internal website (http://bioaqua1.microaqua.eu/signin):

Prerequisite: before proceeding to create a *Microarray Experiment* record, it is required to save all oligos, utilized in the microarray experiment, in MicroAqua database. It can be done manually (from *Oligo Sequence* section) or by utilizing "*Batch file upload*" functionality (refer to the help guide).

All Microarray associated modules come under "*Microarray Experiment*" section, consisting of sections: Microarray GAL, Microarray GPR, Microarray Image, Microarray Analysis and Microarray Validations.

The Microarray Experiment section displays records of individual experiments performed on samples (from Filter Sample table) utilizing oligos (from Oligo Sequence table). One microarray experiment uses one .gal file, providing information about spots positioning on array chip, and produces image files and one result file, the .gpr file.

The microaqua internal website,backed by a database, supports the management of the data files (both .gal and .gpr) and images produced from the scanner.

This document will explain how to handle the data files using MicroAqua internal website.

1. Microarray Experiment section displays records showing unique experiment ID, unique GAL and GPR code, partner code, date of microarray experiment and scanner image associated with the particular experiment.

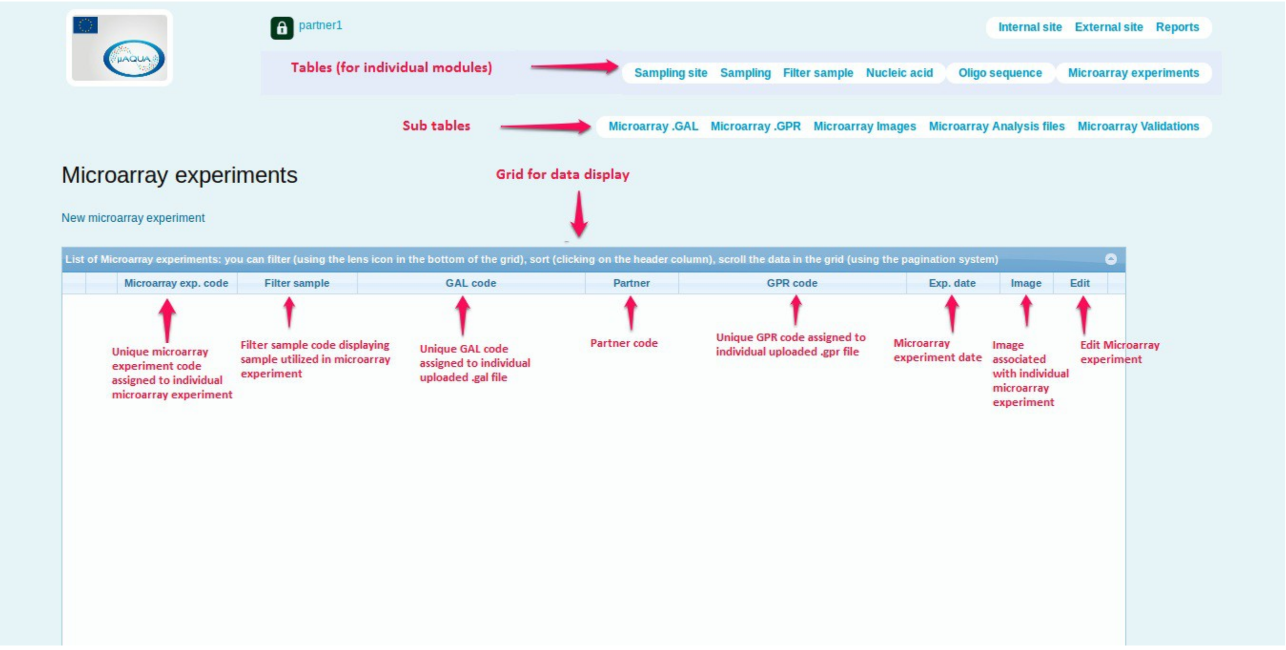

2. To create a microarray experiment record, the related GAL file must first be uploaded. Everytime a new file is uploaded, the resulting page will ask user to upload the other related files. Once the files are uploaded and automatically parsed in the database, the entries are available and must be associated with each other in the "New Microarray Experiment" page.

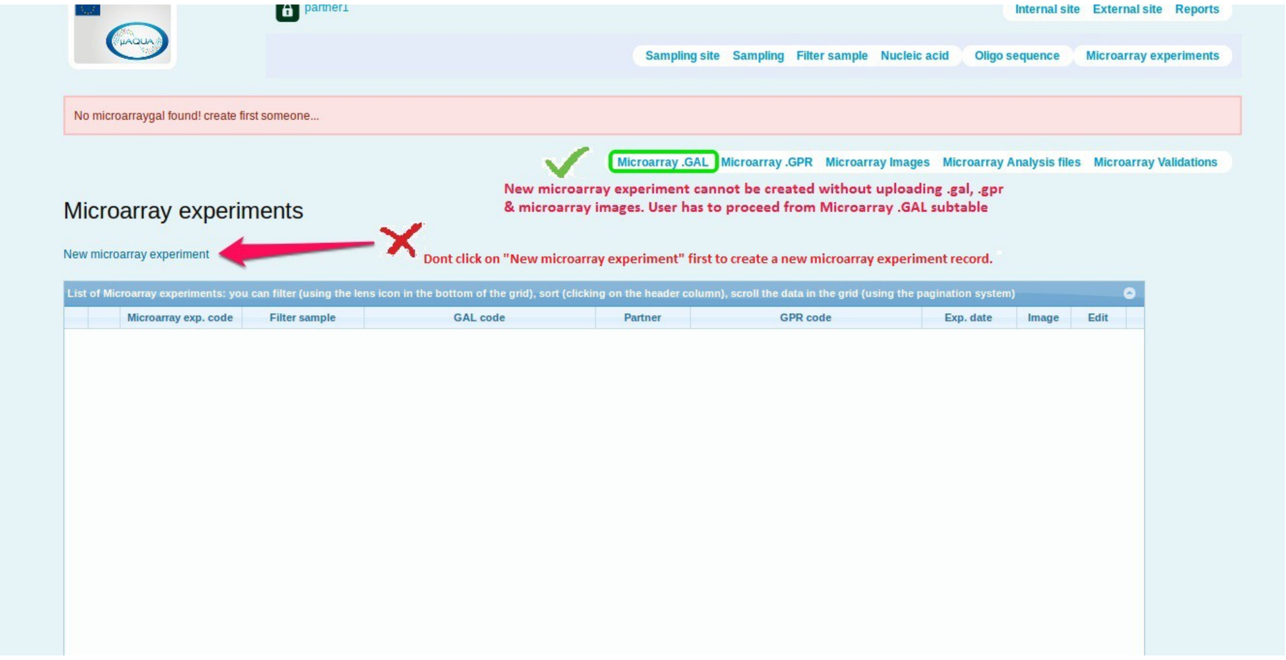

3. Click on the "New microarraygal" link.

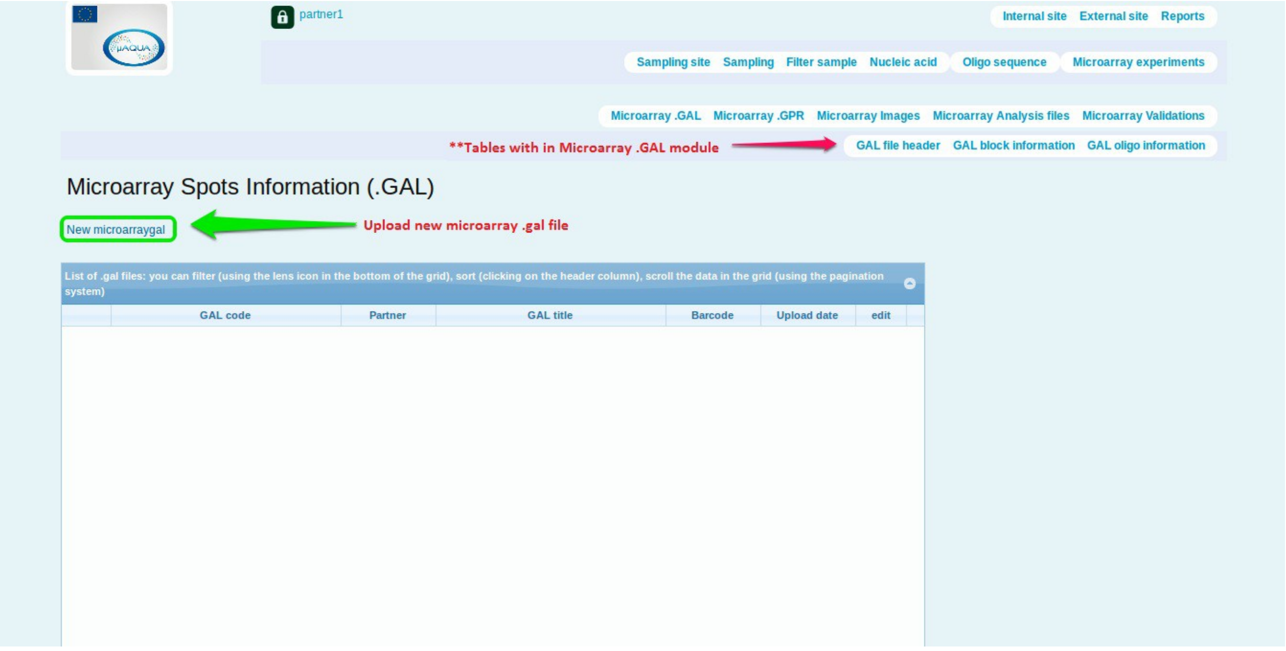

4. Provide a partner barcode (internal name, if any), browse and upload a file. Once the file is uploaded, data are automatically parsed and loaded in the MicroAqua database. The "New microarraygpr" page will pop up to aid in .gpr upload. Follow the same procedure for uploading .gpr file and then for microarray image files produced from GenePix software, modified from Tiff image obtained from the scanner.

(i)

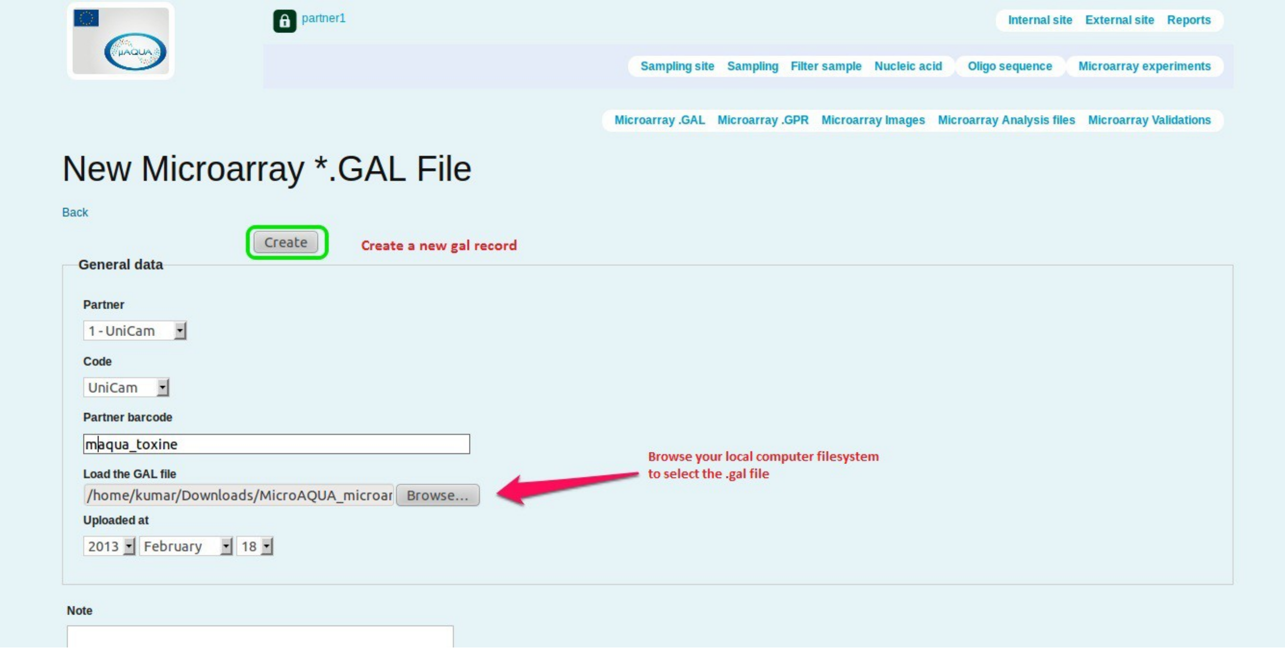

(ii)

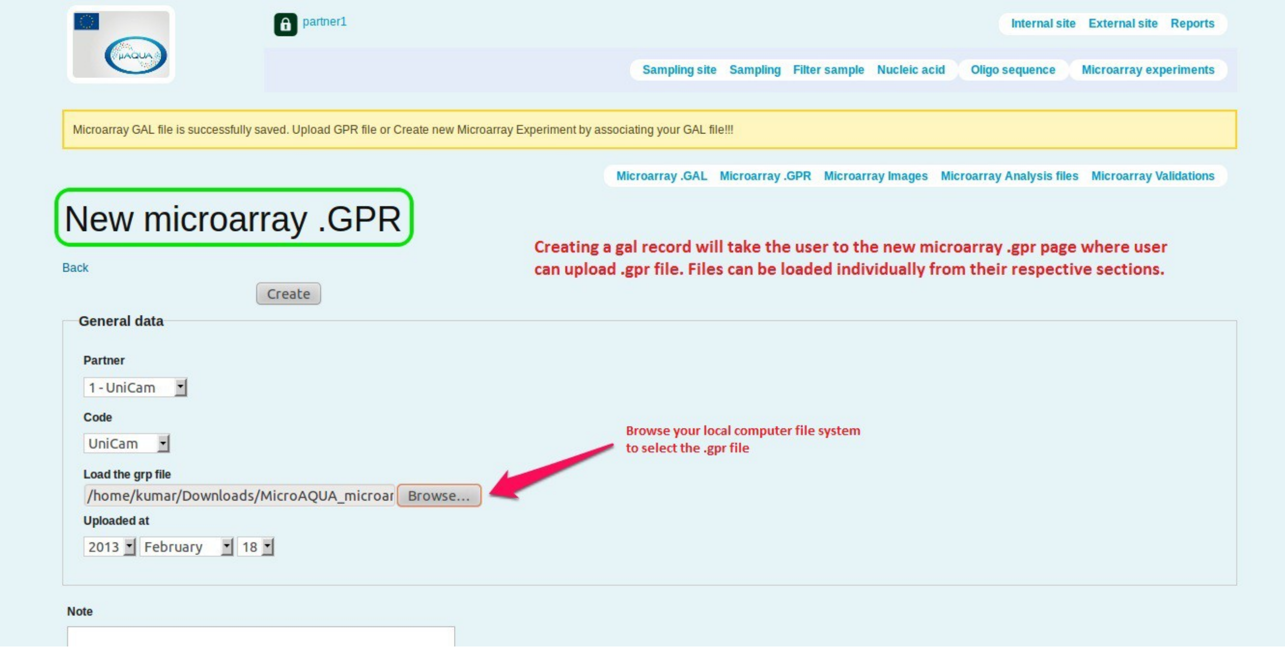

5. On the "New Microarray image" page, user has to fill the relevant information about images and then click "Upload images". If clicked once, one file upload tag will appear. If clicked twice, two upload tags will appear and so on. Maximum three microarray images can be uploaded.

The basename of the image file will be utilized in the unique image code for individual image records.

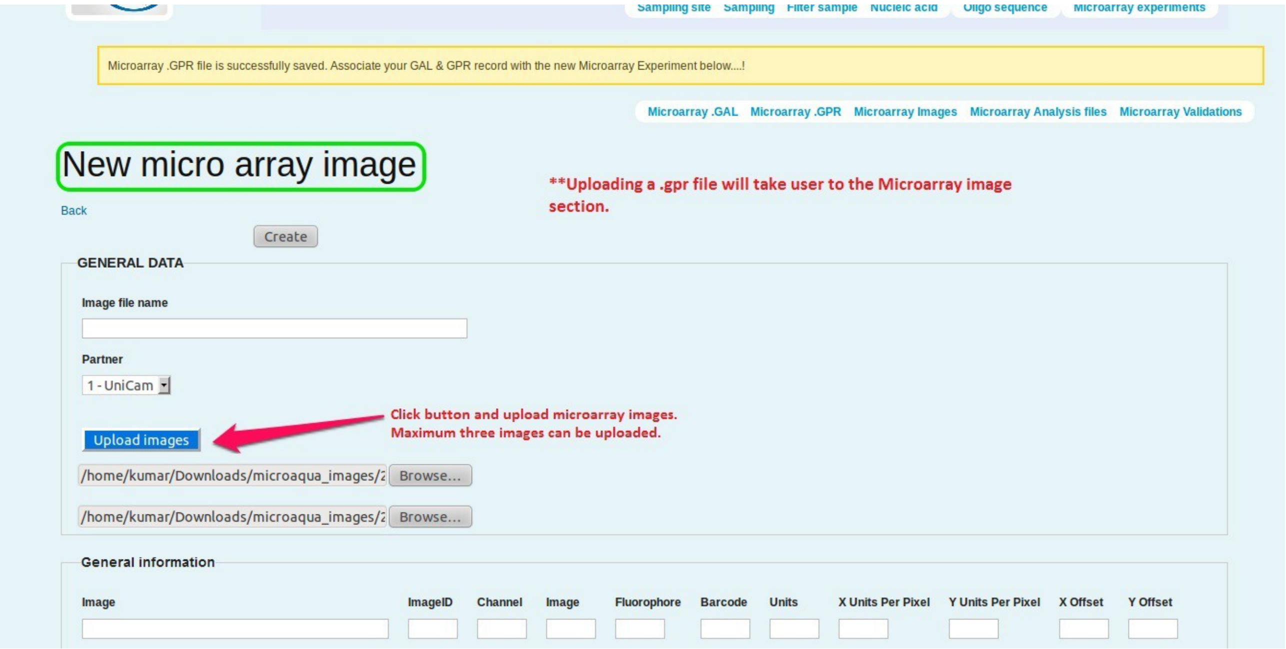

6. Once the image file upload is finished, the page will be directed back to the "New Microarray Experiments" page where all the uploaded files can be associated together in one microarray experiment. Also, the sample utilized (from filter sample) can be associated with the performed microarray experiment.

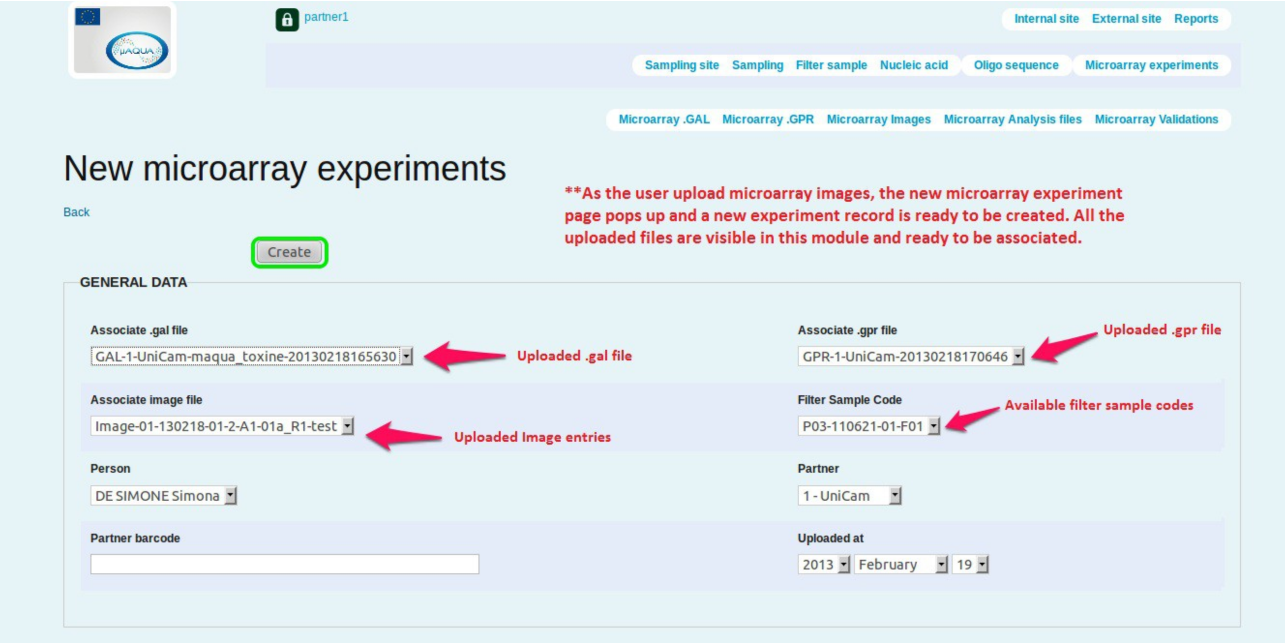

7. Once the "Create" button is clicked, a new record is created for a microarray experiment, displaying all the associated files together in one row, on the index page of "Microarray Experiment" section.

The oligos utilized in this microarray experiment can be seen by clicking on the "+" sign, available at the begining of each row.

The small displayed microarray image can be magnified by clicking on it. Generally, the displayed image is the first image of the "Microarray image" entry.

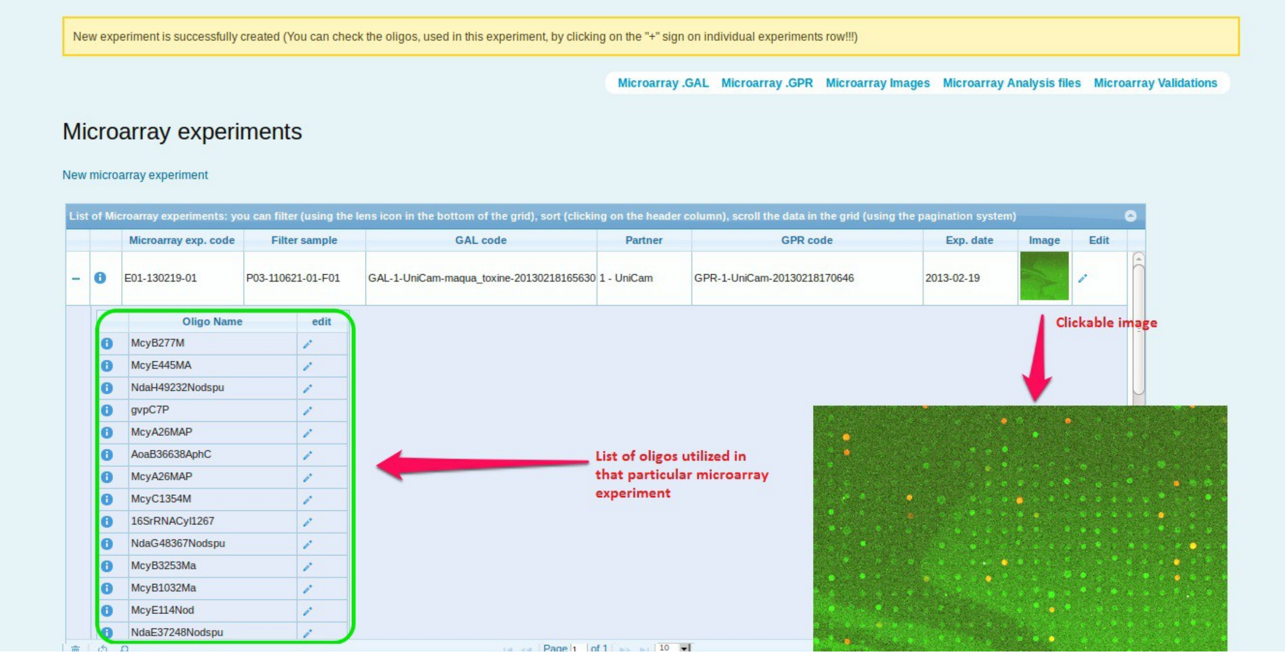

8. The data parsed from the file are available in the sections under Microarray Experiment (Sub tables). Microarray GAL index page displays unique gal code, assigned to individual uploaded file, which differentiates between gal files.

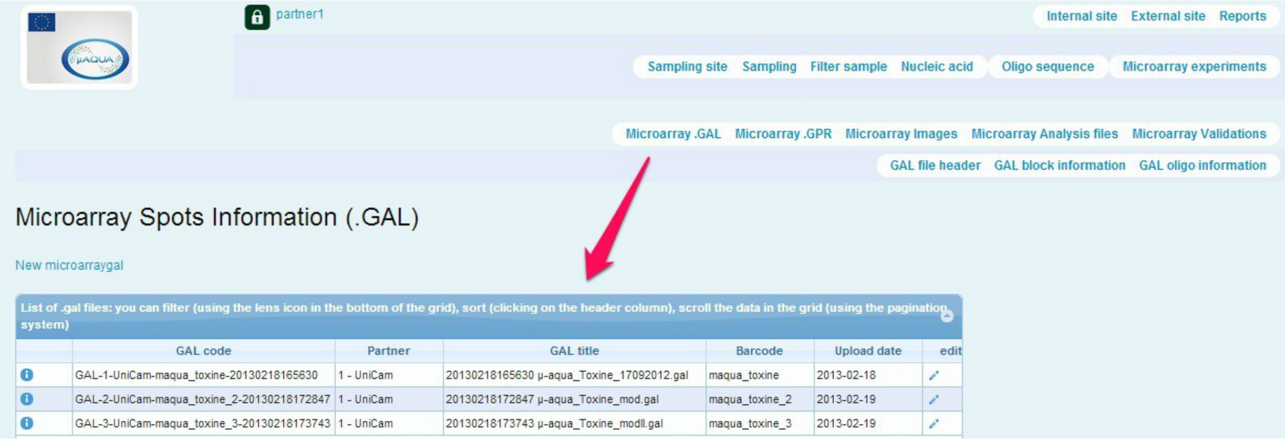

9. The header, block and spot coordinates data are available in sub tables of "Microarray GAL". These data can be edited by clicking on the last column of individual record.

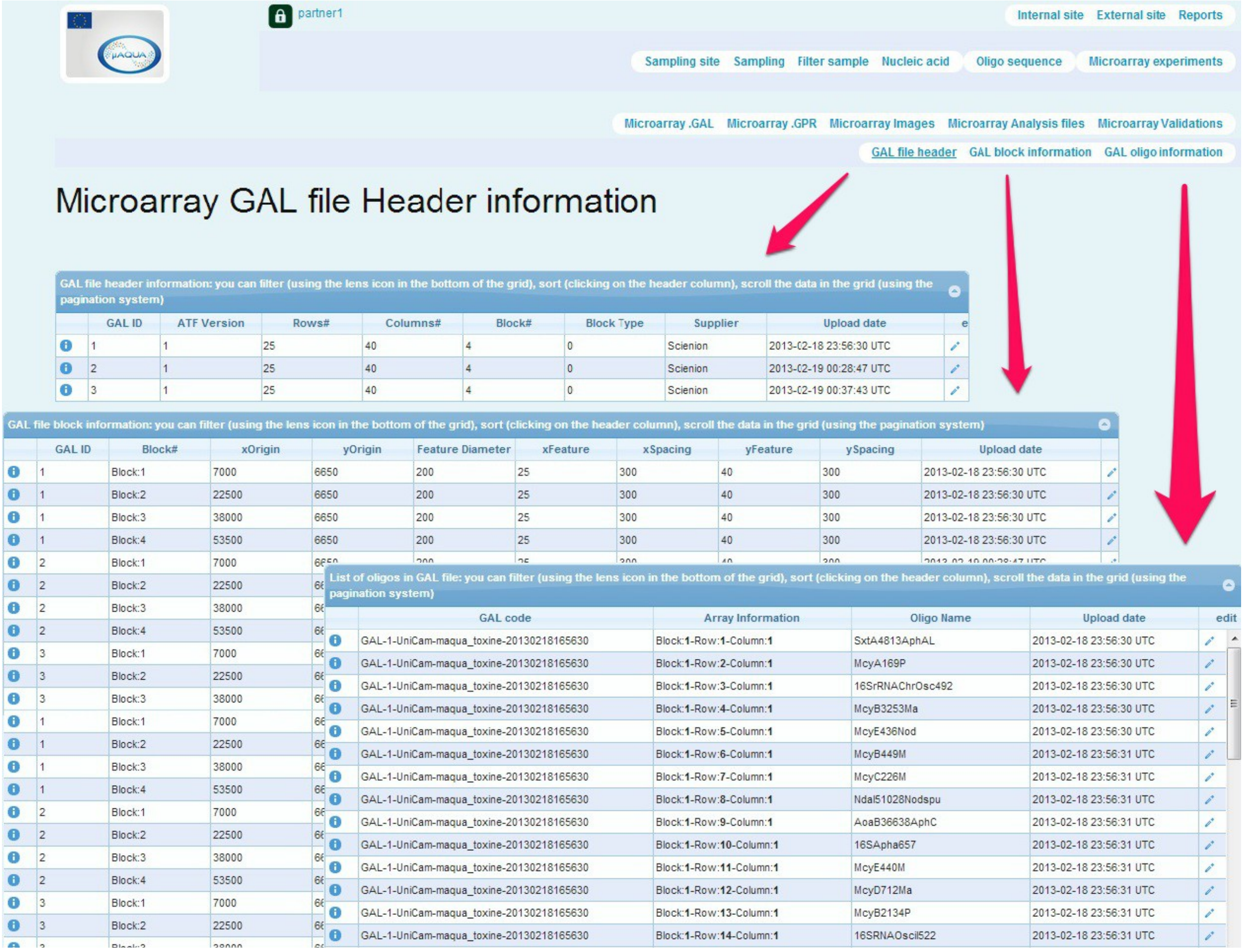

10. The .gpr file is a result of analysis performed on scanner image files and consist of spot intensity data. The raw .gpr file, like .gal file, can be divided in to two sections, the header and the data. These information is available in different sub tables of "Microarray GPR".

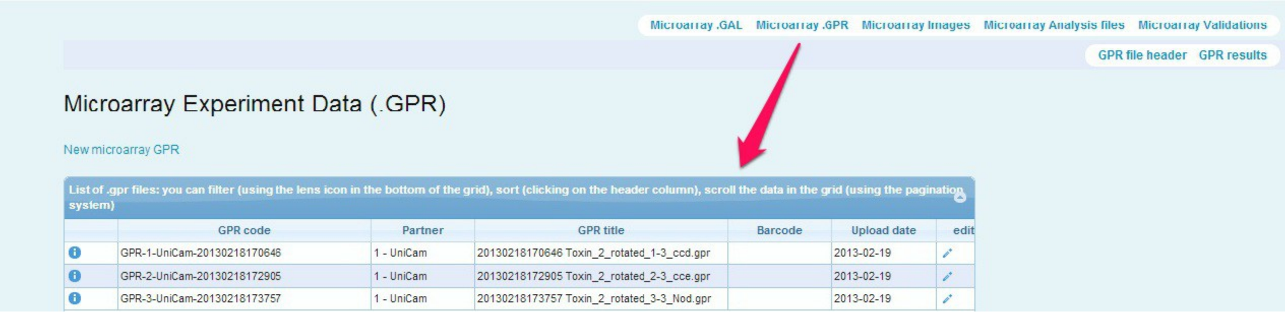

11. The data available/displayed can also be edited and modified.

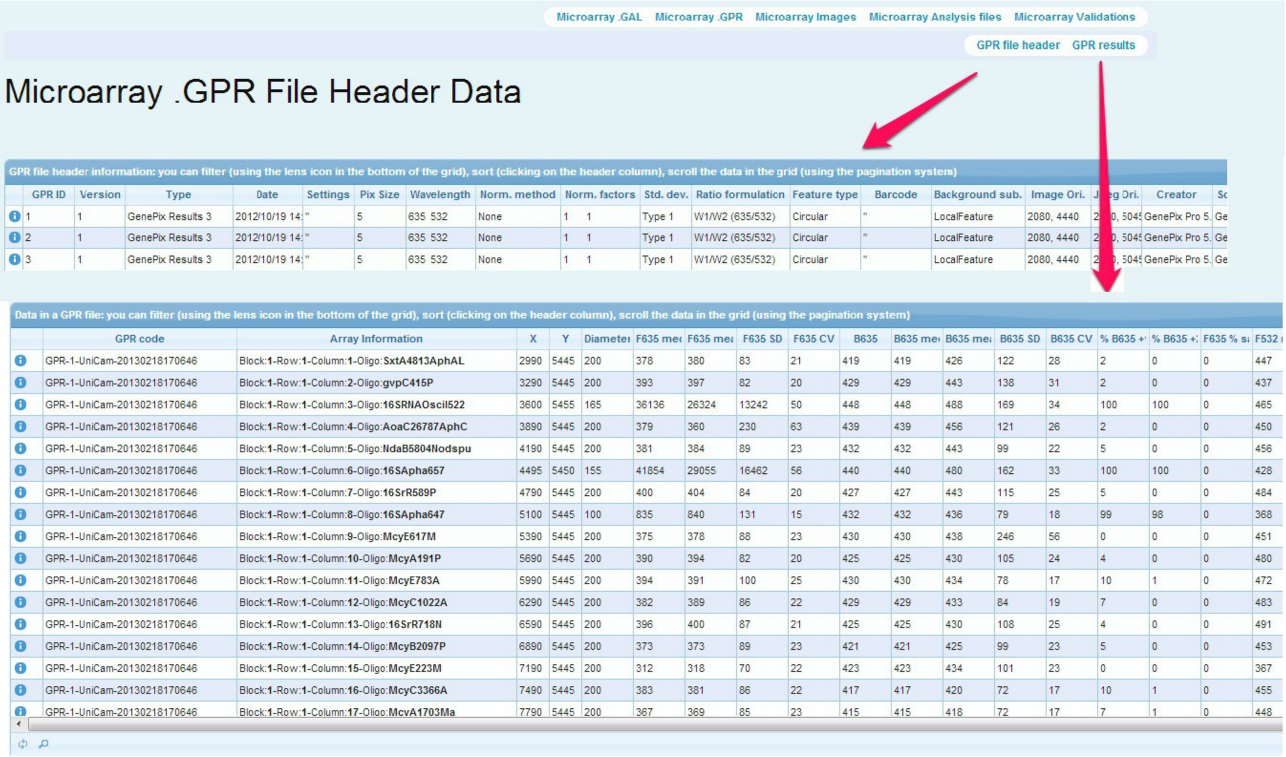

NOTE: The uploaded files can downloaded at anytime from the microaqua server by clicking on the first column of the grid/index which usually displays all the information related to that entry.

12. The index page of the "Microarray Image" section displays unique code generated for indivdual entry and also shows other related information. Each individual image entry can have maximum 3 images, available from the "Details" column (first column of the grid). The images can be replaced and entries can be modified from the "Edit" column.

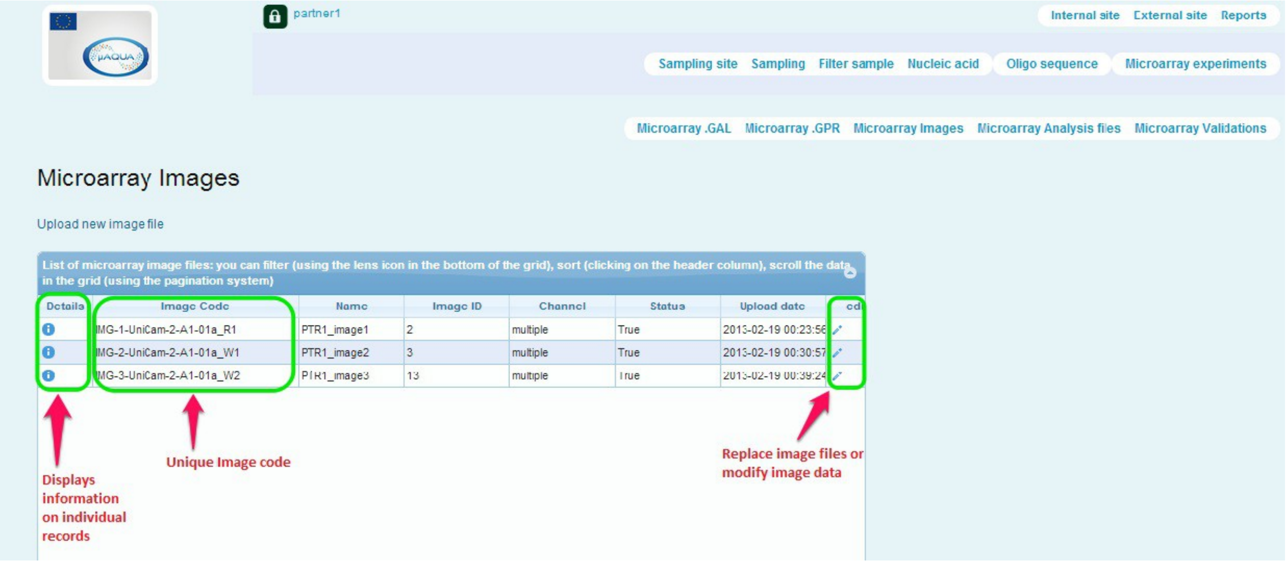

Supplement: Supplementary file 1 [file ijerph-12-06352-s001.zip › 6_Microarray_experiment.pdf]
