# Supplementary material for: SaDA: From Sampling to Data Analysis—An Extensible Open Source Infrastructure for Rapid, Robust and Automated Management and Analysis of Modern Ecological High-Throughput Microarray Data
Source: Int J Environ Res Public Health. 2015 Jun 3;12(6):6352–66. doi: 10.3390/ijerph120606352 (PMC4483705; doi:10.3390/ijerph120606352)
Supplement: Supplementary file 1 [file ijerph-12-06352-s001.zip › 7_Microarray_analysis.pdf]

Microarray Analysis Section

Once all the raw data files are associated under Microarray Experiment Section of SaDA, the analysis pipeline can be initiated.

User associates all the data together in one section.

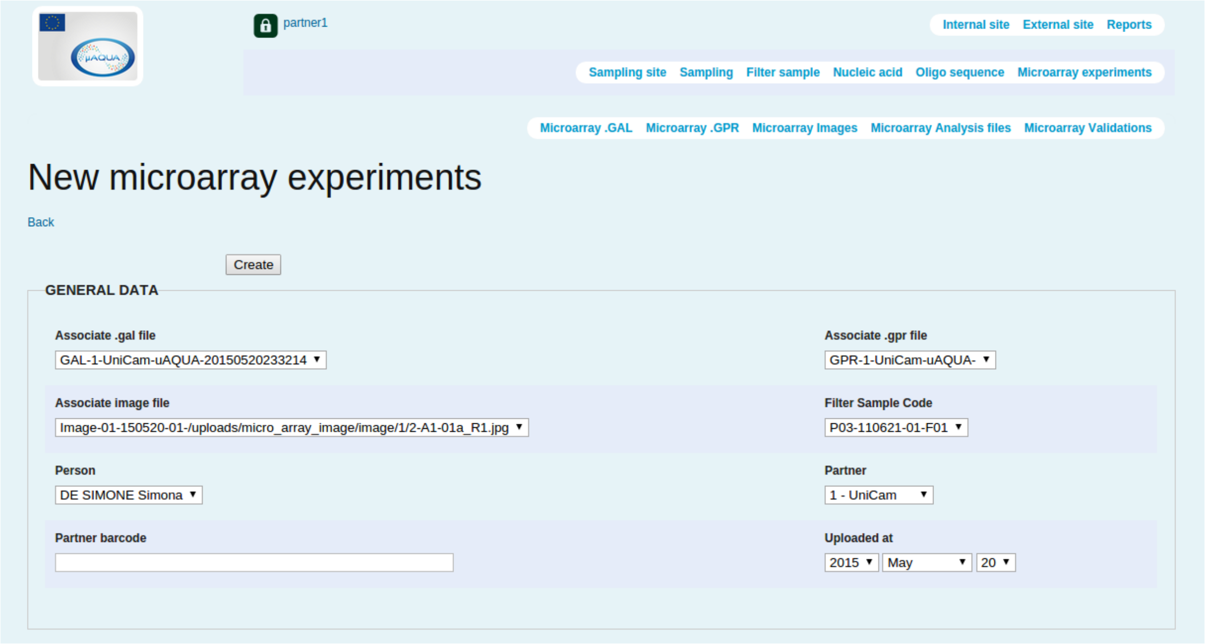

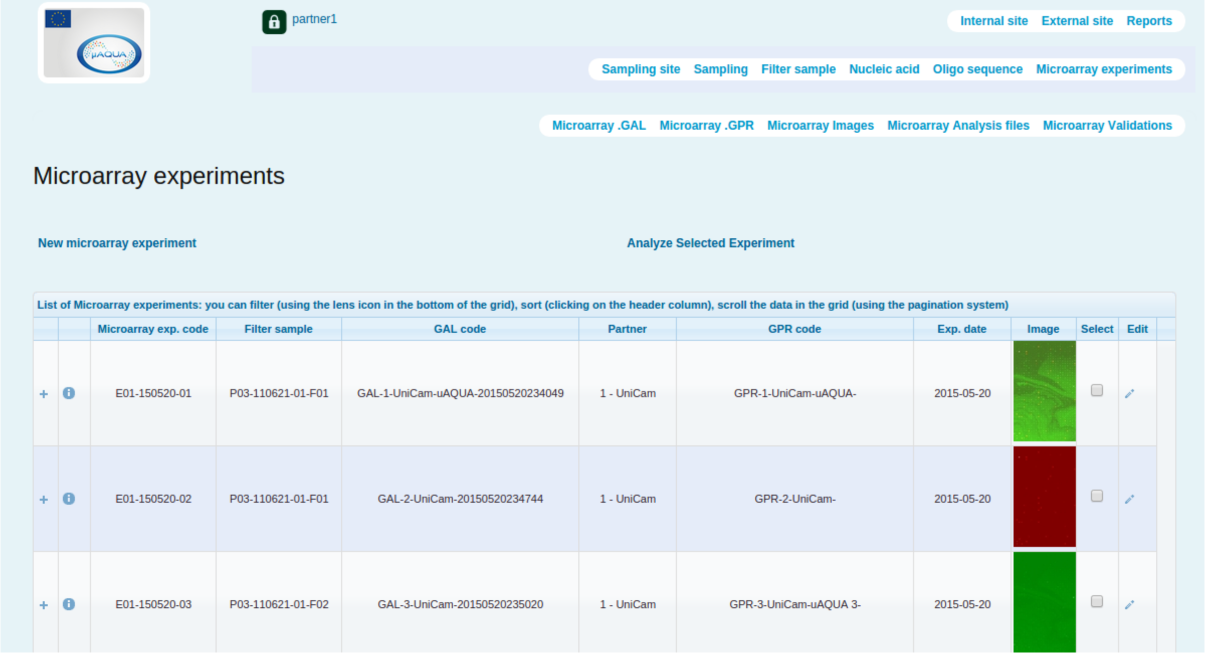

The hybridization experiments that need to be analysed are checked. User can then click the link titled "*Analyse Selected Experiment*".

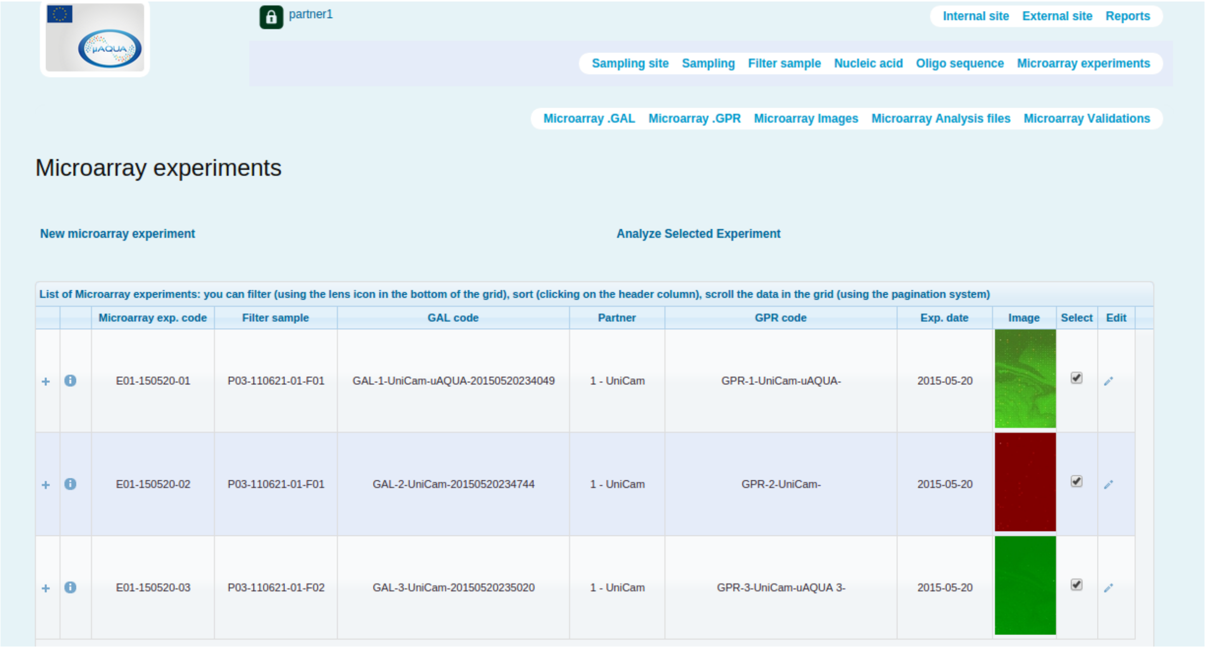

With the click, SaDA sends all the raw data to R programming environment for its further analysis. SaDA calculates signal to noise ratio (SNR) and Total Signal Intensity (TSI) for all the spotted probes. The resulting data is displayed under *Microarray Analysis* section. This analysed secondary data can be exported in CSV and XLS format.

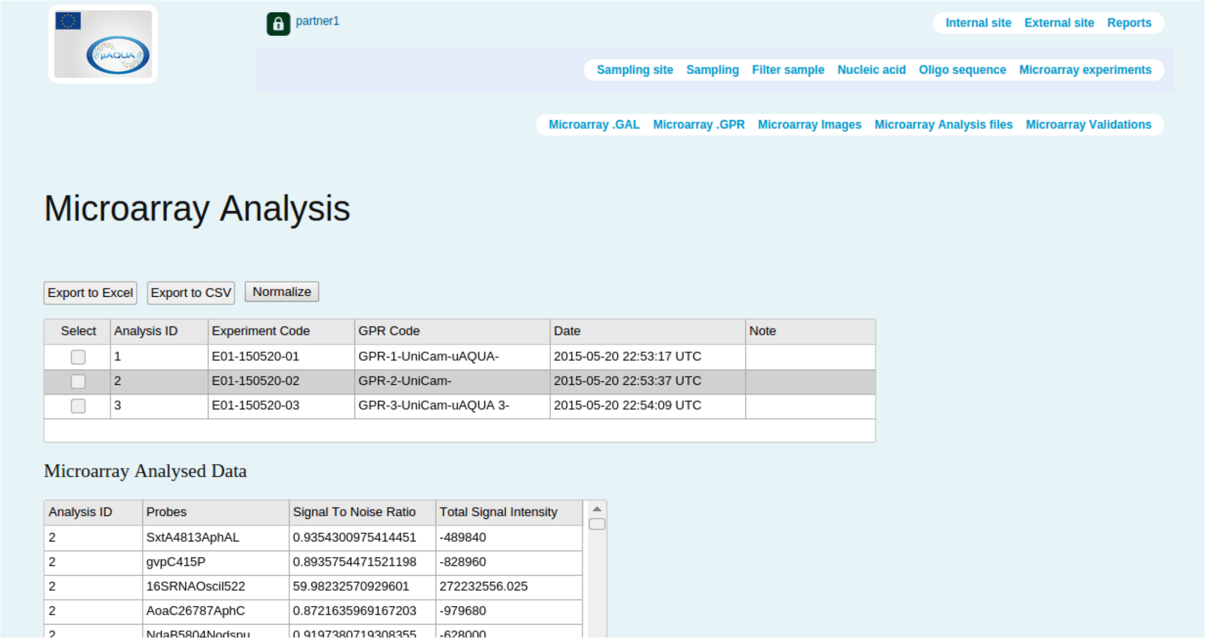

In the Microarray Analysis section two data tables are visible. Upper table displays the experiment metadata, like the experiment code, GPR file code, analysis unique ID and date of creation. When user hovers over and clicks on individual rows of table 1, the data in table 2 changes. So for every experiment and generated GPR file there is one analysed data with calculated SNR and TSI for every spotted probe. User can also select on individual row in table 1 and click on Normalize. This action will normalize the SNR and TSI data based on individual control probe intensity. Probe selection menu will appear once the user clicks on Normalize button. SaDA also renders images for normalized

probes hybridization value, generated by the R software and it creates bar charts using jQwidgets charting library.
